# Comparative metabolites profiling of different solvent extracts of *Asparagus* species cladodes using liquid chromatography–mass spectrometry‐based metabolomics and molecular networking

**DOI:** 10.1002/pca.3446

**Published:** 2024-09-09

**Authors:** Pfano W. Maphari, Mthokozisi B. C. Simelane, Ntakadzeni E. Madala, Msizi I. Mhlongo

**Affiliations:** ^1^ Department of Biochemistry, Faculty of Science University of Johannesburg Auckland Park Gauteng South Africa; ^2^ Department of Biochemistry and Microbiology, Faculty of Science, Engineering and Agriculture University of Venda Thohoyandou Limpopo South Africa

**Keywords:** *Asparagus*, different solvents, LC–MS, metabolomics, molecular networking, phytochemical compounds

## Abstract

**Introduction:**

*Asparagus* species are naturally distributed worldwide and are known for their pharmacological properties that offer cures for various ailments. However, the metabolic choreography of these *Asparagus* species is not well characterized, and the compounds contributing to their bioactivities remain unknown.

**Objective:**

This study aimed to profile and compare the metabolomes of three *Asparagus* species cladodes using different solvent extractions.

**Methods:**

An ultra‐high performance liquid chromatography–quadrupole time‐of‐flight mass spectrometry‐based metabolomics and molecular networking approach was used to study the effects of different solvents (ethyl acetate, methanol, and chloroform) with varying polarity on metabolites extraction and identification of bioactive compounds from three *Asparagus* species cladodes ( *Asparagus falcatus* , *Asparagus plumosus* , and *Asparagus densiflorus* ‘Meyersii’).

**Results:**

Multivariate statistical analyses (mainly principal component analysis) revealed a significant separation between the three solvents and the three species, indicating notable metabolic differences. A total of 118 metabolites were identified in the three species extracted with the different solvents, with methanolic and chloroform extracts containing more metabolites compared with ethyl acetate extracts. These metabolites were identified as belonging to the flavonoids, cinnamic acids, organooxygen compounds, steroids, fatty acids, benzenes, and glycerophospholipids compound classes. Furthermore, these compounds classes were differentially distributed among the three species, indicating chemical/chemotaxis differences between the compared species. Chloroform and methanol are recommended as the optimal solvents to obtain a high content of phytochemical compounds from *Asparagus* species cladodes.

## Abstract 2

The study profiled metabolites from three Asparagus species using different solvents (ethyl acetate, methanol, and chloroform) with LC‐MS‐based metabolomics and molecular networking. A total of 118 metabolites, including flavonoids, cinnamic acids, steroids, and glycerophospholipids, were identified. Chloroform and methanol were more effective in extracting metabolites compared to ethyl acetate. The study also revealed metabolic differences between the species, which align with their taxonomic classification, as each species exhibited unique metabolite profiles, underscoring their distinct chemotaxonomic identities and potential medicinal applications.

## 1 INTRODUCTION

*Asparagaceae* is a large and diverse family of flowering plants, with about 2900 known species. The genus *Asparagus* is a herbaceous plant from the family *Asparagaceae* with approximately 300 species that are naturally distributed.^1^, ^2^ This genus is widely used as a healthy food supplement with valuable contents and for medicinal purposes due to its phytochemical constituents.^3^ The major bioactive compounds found in *Asparagus* are flavonoids, hydroxycinnamic acids, and steroidal saponins, which contribute to the antimicrobial, antimalarial, anticancer, antibacterial, and anti‐inflammatory properties of the genus.^4^, ^5^ Some of the species within this genus include *Asparagus falcatus*, *Asparagus plumosus*, and *Asparagus densiflorus* ‘Meyersii’, which will be the main focus of this study.

*A. falcatus*, commonly known as the sicklethorn or large forest *Asparagus*, is characterized by its distinctive sickle‐shaped cladodes and is native to southern Africa. *A. falcatus* thrives in well‐drained soil and is often found in regions with moderate temperatures and ample rainfall.^6^ Traditionally, various parts of *A. falcatus* have been utilized for their medicinal properties in African folk medicine, particularly for ailments such gonorrhea and hernias.^7^ Additionally, extracts from *A. falcatus* have shown potential in preliminary studies for their antioxidant and antimicrobial properties, indicating promise for pharmaceutical applications.^8^ *A. plumosus*, also known as the climbing *Asparagus* fern, is native to South Africa and is known for its delicate, feathery cladodes. *A. plumosus* is adaptable to a variety of habitats but is commonly found in coastal regions, forests and grasslands.^9^ It has been traditionally used in medicine; its extracts have demonstrated antibacterial and antifungal activities, suggesting potential therapeutic applications.^9^, ^10^ *A. densiflorus* ‘Meyersii’ commonly known as the foxtail fern is characterized by its dense, upright growth habit and needle‐like foliage. This cultivar of *A. densiflorus* is well‐suited to both indoor and outdoor environments and flourishes in partial to full shade and is often used as a decorative plant in gardens and landscapes.^11^, ^12^ Although primarily cultivated as an ornamental plant, *A. densiflorus* ‘Meyersii’ has attracted attention for its potential medicinal properties. Research indicates that extracts from this species possess antioxidant and anti‐inflammatory properties, which could be explored further for pharmaceutical purposes.^11^, ^12^ Although researchers have been investigating the chemical composition of *Asparagus* species, the chemical space of these species is not well decoded; their bioactive compounds are thus unknown. This study therefore explored the metabolomic composition and outlines compounds that contribute to the bioactivity of the *Asparagus* species cladodes.

Metabolomics has become an effective technique for various applications, such as toxicity, quality control, natural product research, and disease studies.^13^, ^14^ Recently, there has been a surge of interest in the use of metabolomics to analyze the constituents of natural products. The goal of metabolomics is to detect and quantify small molecules in a biological system, as well as to measure the changes in the metabolite profile due to environmental perturbations.^15^, ^16^ Metabolomics has emerged a potent chemotaxonomic method that provides a thorough understanding of the chemical differences and similarities among medicinal plants.^17^, ^18^ This approach has primarily been applied to plant species and subspecies, allowing for the differentiation of closely related species.^19^, ^20^

To successfully apply metabolomics to resolve phylogeny, suitable sampling and analysis methods should be selected. Metabolite extraction is a crucial step in metabolomics, as it affects the metabolite features detected and the biological interpretation of the resulting data. Therefore, choosing an efficient and reliable metabolites extraction and sample preparation procedure is extremely important.^21^ Furthermore, the appropriate extraction method should be fast and simple to ensure the reproducibility of accurate metabolome coverage.^22^ Solvent extraction is one of the most common extraction methods and takes advantage of differential solvent solubility and immiscibility.^23^ Various solvents, including methanol, ethanol, and acetone, have been used to extract polar bioactive compounds while also increasing the solubility of less‐polar metabolites.^15^, ^24^ Additionally, non‐polar organic solvents such as chloroform, ethyl acetate, and hexane are often paired with polar solvents to enable the separation of both polar and non‐polar metabolites.^25^ Both polar and non‐polar solvents have been used to extract chemo‐diverse metabolites and to increase metabolome coverage.^15^, ^26^, ^27^

In this regard, the metabolites composition of plants is highly linked to the type of solvents/method used for extraction.^28^, ^29^ Because the primary goal of the extraction process is to extract a wide variety of chemo‐diverse compounds from the sample, the choice of extraction solvent is thus a crucial component factor in identifying the metabolites of interest.^29^, ^30^ To obtain a broad spectrum of metabolites, solvents are chosen to match the polarity of the metabolites of interest.^31^

Given the chemodiversity of metabolites found in medicinal plants, various analytical methods have been developed to achieve a comprehensive coverage of the plant metabolome. Among these analytical tools, liquid chromatography–mass spectrometry (LC–MS) has shown advantages over other methods, such as gas chromatography–mass spectrometry (GC–MS) and nuclear magnetic resonance (NMR).^32^, ^33^ Moreover, the results obtained by the LC–MS are extremely multidimensional and require complex statistical models that allow the decoding of the underlying patterns.^34^ Principal component analysis (PCA) and partial least squares discriminant analysis (PLS‐DA) models are examples of multivariate statistical analysis methods that have been proven to be effective.^35^, ^36^ Molecular network is another metabolomics tool used to classify and identify similarity between metabolites.^37^ MN groups compounds into molecular families based on fragmentation similarities and searches MS^2^ spectra libraries for annotation. Annotating molecules in the molecular cluster can aid in the annotation of structurally related compounds.^38^, ^39^ Other GNPS ecosystem tools (i.e., Network Annotation Propagation [NAP], Dereplicator, and MS2LDA) are used to visualize chemical classes and the variety of substructures within the molecular families.^40^, ^41^

Therefore, the current study employed an LC–MS‐based metabolomics approach to profile the metabolites of three *Asparagus* species cladodes (*A. falcatus*, *A. plumosus*, and *A. densiflorus* ‘Meyersii’) extracted with chloroform, ethyl acetate, and methanol. In addition, a detailed chemotaxonomic comparison of *Asparagus* species was conducted using the Global Natural Product Social Molecular Networking (GNPS) approach.

## 2 MATERIALS AND METHODS

### 2.1 Plant cultivation

Seeds of three *Asparagus* plant species (*A. falcatus*, *A. plumosus*, and *A. densiflorus* ‘Meyersii’) were purchased from Seeds of Africa. The seeds were sowed in 4 L plastic bags filled with potting soil and placed in a growth chamber with the following conditions: minimum temperature of 15°C and maximum temperature of 25°C, light/dark cycle of 12/12 h, and light intensity of 60 μmol/m^2^/s. The seedlings were watered as needed and Vita Veg organic fertilizer (Talborne Organics, South Africa) was applied post‐germination. The plants were grown for 4 months before harvesting (Figure S1).

### 2.2 Metabolite extraction

After 4 months, the cladodes of *A. falcatus*, *A. plumosus*, and *A. densiflorus* ‘Meyersii’ (Figure S1) were harvested, freeze‐dried, and blended in a blender into a fine powder. Fifty milligrams of the powdered cladodes was extracted with 1.5 mL of various solvents (ethyl acetate, methanol, and chloroform). The samples were shaken at 70 rpm overnight for efficient extraction. Cell debris was removed by centrifugation at 8000 rpm for 10 min. The supernatant was transferred into a clean 2 mL Eppendorf tube and dried to completeness at 55°C in a speed vacuum microcentrifuge. The dried residues were reconstituted in 500 μL 50% methanol and filtered through a 0.22 μm nylon filter into a 2 mL vial fitted with a 500 μL conical insert. The filtered extracts were stored at −20°C until analysis.

### 2.3 LC–MS analysis

The extracts were analyzed using an ultra‐high performance liquid chromatography–quadrupole time‐of‐flight mass spectrometry (UHPLC‐qTOF‐MS) (Shimadzu Corporation, Kyoto, Japan) model 9030 instrument fitted with a Shim Pack Velox Gist C18 column (100 mm × 2.1 mm, 2.7 μm particle size) (Shimadzu Corporation, Kyoto, Japan) placed in a column oven thermostatted at 40°C. A binary solvent mixture consisting of 0.1% formic acid in water (Eluent A) and 0.1% formic acid in methanol (Eluent B) was used at a constant flow rate of 0.3 mL/min and a total run time of 30 min. The gradient for Eluent B ranged from 2% over 0.0 to 1.0 min, 2% to 60% over 2.0 to 24 min, 60% to 95% over 24 to 25 min, and from 25 to 27 min, the conditions were maintained at 95% and returned from 95% to 2% over 27 to 28 min. The data was acquired in negative ionization mode. The conditions of the MS were set as follows: interface voltage of 4.0 kV, interface temperature of 300°C, heat block temperature of 400°C, desolvation line temperature of 280°C, detector voltage of 1.8 kV, and the flight tube temperature at 42°C MS1 and MS2 (through data‐dependent acquisition) was generated simultaneously for all ions with an *m/z* range between 100 and 1000 surpassing an intensity threshold of 5000. Quality control samples were used to assess the reliability, reproducibility, and non‐linear signal correction. Sample data acquisition was randomized, and the quality control sample was analyzed every five injections to monitor and correct changes in the instrument response.

### 2.4 Multivariate data analysis

After data acquisition, the raw data files were converted to the mzML format and further used for multivariate analysis using an open‐access web‐based metabolomic data processing tool called MetaboAnalyst 5.0 (https://www.metaboanalyst.ca/). Data processing involved checking data integrity and missing value estimation, normalization by median, and log transformation, followed by Pareto scaling of datasets. After normalization and data processing, the data were analyzed using PCA.

### 2.5 GNPS and metabolite annotation

A molecular network was created on the GNPS website (http://gnps.ucsd.edu) using the online workflow (https://ccms-ucsd.github.io/GNPSDocumentation/). Raw data from the ShimadzuLCMS‐9030 qTOF‐MS were initially converted to mzML and uploaded to WinSCP, which store and transfer the data online. Once the data were uploaded, the precursor ion mass tolerance was set at 2.0 Da and MS/MS fragment ion tolerance at 0.5 Da. Edges were formed only if the minimum cosine score of 0.7 was exceeded with more than six matched peaks. The maximum number of nodes that can be connected into a single molecular family was set at 100 and the lowest‐scoring edges were removed. The output of the molecular network was visualized using Cytoscape version 3.9.0. MolNetEnhancer workflow was used to enhance structural annotations by integrating the generated networks with Dereplicator+, MS2LDA, and NAP by enabling structural searches based on the databases: GNPS, Human Metabolome Database (HMDB), SuperNatural (SUPNAT), Chemical Entities of Biological Interest (CHEBI), DRUGBANK, and Food Database (FooDB). For compound annotations, all matched and unmatched nodes were verified using the empirical formulas generated from accurate mass and fragmentation patterns obtained from the MS/MS data (e.g., diferuloyl glycerol [glyceryl 1,2‐diferulate] identified with the parent mass 443.176 [M‐H], C_23_H_24_0_9_, and the product ions at *m/z* 249.36, 193.10, and 134.03). This was applied to all identified metabolites as presented in Table S1. These compounds were also compared with other common natural product dereplication databases such as KNApSAck (www.knapsackfamily.com), PubChem ( pubchem.ncbi.nlm.nih.gov), and ChemSpider (www.chemspider.com). The compounds were also confirmed using Sirius software.

### 2.6 Pathway analysis and relative quantification

Annotated metabolites were used for pathway analysis using a web‐based tool in MetaboAnalyst 5.0 called Metabolomic Pathway Analysis (MetPA) (http://metpa.metabolomics.ca). Compound names were used as input for pathway analysis with the following parameters: Hypergeometric test was used as enrichment method for overrepresentation analysis, relative centrality was chosen to analyze the topology of node importance, a scatter plot was used for visualization, and *Arabidopsis thaliana* (KEGG) was selected as the path library. MetaboAnalyst was used to generate color‐coded heatmaps for relative quantification, where the highest intensity table was imported and the uploaded data were normalized, Pareto scaled, and log transformed. Other defined parameters included the use of Ward's clustering algorithm and Pearson's correlation. To simplify the visualization of metabolite abundances across the species, the top 25 metabolites are shown in rank by *t* test or analysis of variance (Figure 7A).

## 3 RESULTS

### 3.1 Multivariate data analysis of phytochemicals from different solvent extracts of *Asparagus* species

**FIGURE 1:**
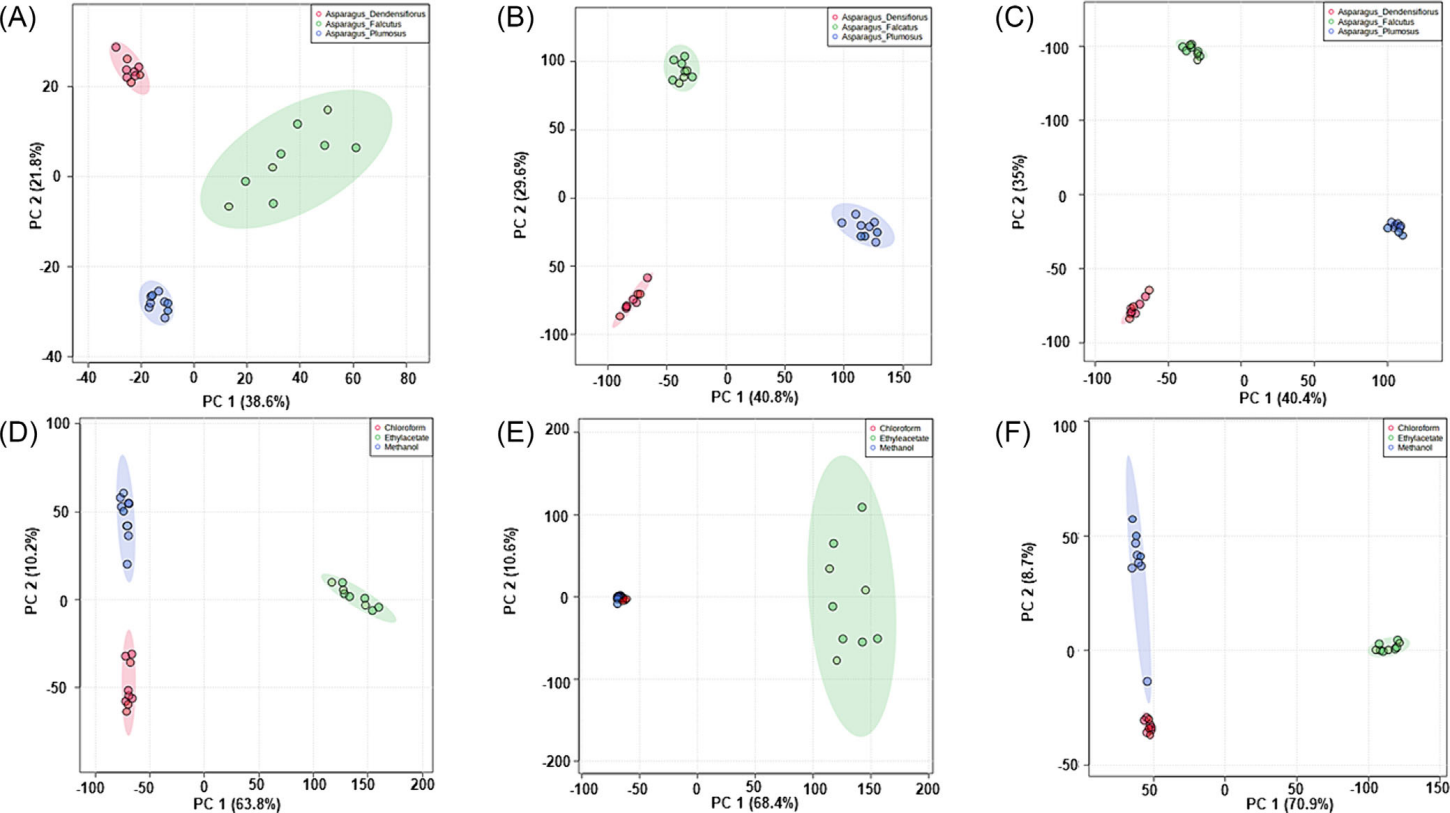
(A–C) PCA score scatterplots indicating differences among different solvents (ethyl acetate [A], methanol [B], and chloroform [C]), where the color code represents samples within *A. densiflorus* ‘Meyersii’ (red), *A. falcatus* (green), and *A. plumosus* (blue). The model obtained was a two‐component model that explained 60.4% (A), 70.4% (B), and 75.4% (C) of the variation. (D–F) PCA score plot of *A. densiflorus* ‘Meyersii’ (D), *A. falcatus* (E), and *A. plumosus* (F). Datasets are initiated from ethyl acetate (green), methanol (blue), chloroform (red), and they indicate different clustering patterns. The model obtained was a two‐component model that explained 74% (D), 79% (E), and 79.6% (F) of the total variation.

In this study, metabolite profiling of the solvent extracts of *Asparagus* species cladodes was conducted by LC–MS/MS using electrospray ionization in negative mode. With LC–MS, variations in the metabolic makeup of samples from various plants/solvents can be visually described. Chromatographic observations indicated that the three *Asparagus* species differed in peak intensities and the presence/absence of peaks and similar observations were made for the solvents (varying peak intensities and presence/absence of peaks) (Figure S2). To determine the metabolomic difference between *Asparagus* species, multivariate analyses were performed. The most practically utilized multivariate analysis technique used to minimize the dispersity of datasets is the unsupervised method, namely, PCA.^42^ The generated two‐dimensional PCA score plot allowed for the assessment and grouping of samples according to the similarities/differences of their metabolic fingerprints (Figure 1). The resulting PCA score plot revealed differential clustering of samples into three distinct groups: *A. densiflorus* ‘Meyersii’ (red), *A. falcatus* (green), and *A. plumosus* (blue) (Figure 1A–C) and ethyl acetate (green), methanol (blue), and chloroform (red) (Figure 1D–F). Based on the observations on the PCA score plots, the samples from each species were clustered together, which indicates that the samples within each species shared common metabolite profiles. Samples of *A. densiflorus* ‘Meyersii’, *A. falcatus*, and *A. plumosus* were observed to cluster separately, reflecting the significant underlying metabolic differences. The PCA results showed that the species occupy distinct regions of the PCA score plot, highlighting their metabolic differences (Figure 1A–C). Furthermore, the comparison of solvents also revealed clear separation of samples, suggesting significant metabolic differences (see Figure 1D–F). Interestingly, the methanol extracts and chloroform extracts were seen to cluster at the same point (Figure 1E). This implies that methanol and chloroform extracted similar metabolic features compared with ethyl acetate.

In addition, another PCA comprising all extracts of the three *Asparagus* species (extracted with three different solvents, separately) was generated to further analyze differences and similarities between the extracts (Figure S3). The results displayed methanol and chloroform samples as closely spaced in a small area (thus related), which suggests that the extraction from these two solvents have similar main components and that the differences in their metabolites are not obvious (Figure S3A).

### 3.2 Molecular networking‐based dereplication of *Asparagus* metabolites

**FIGURE 2:**
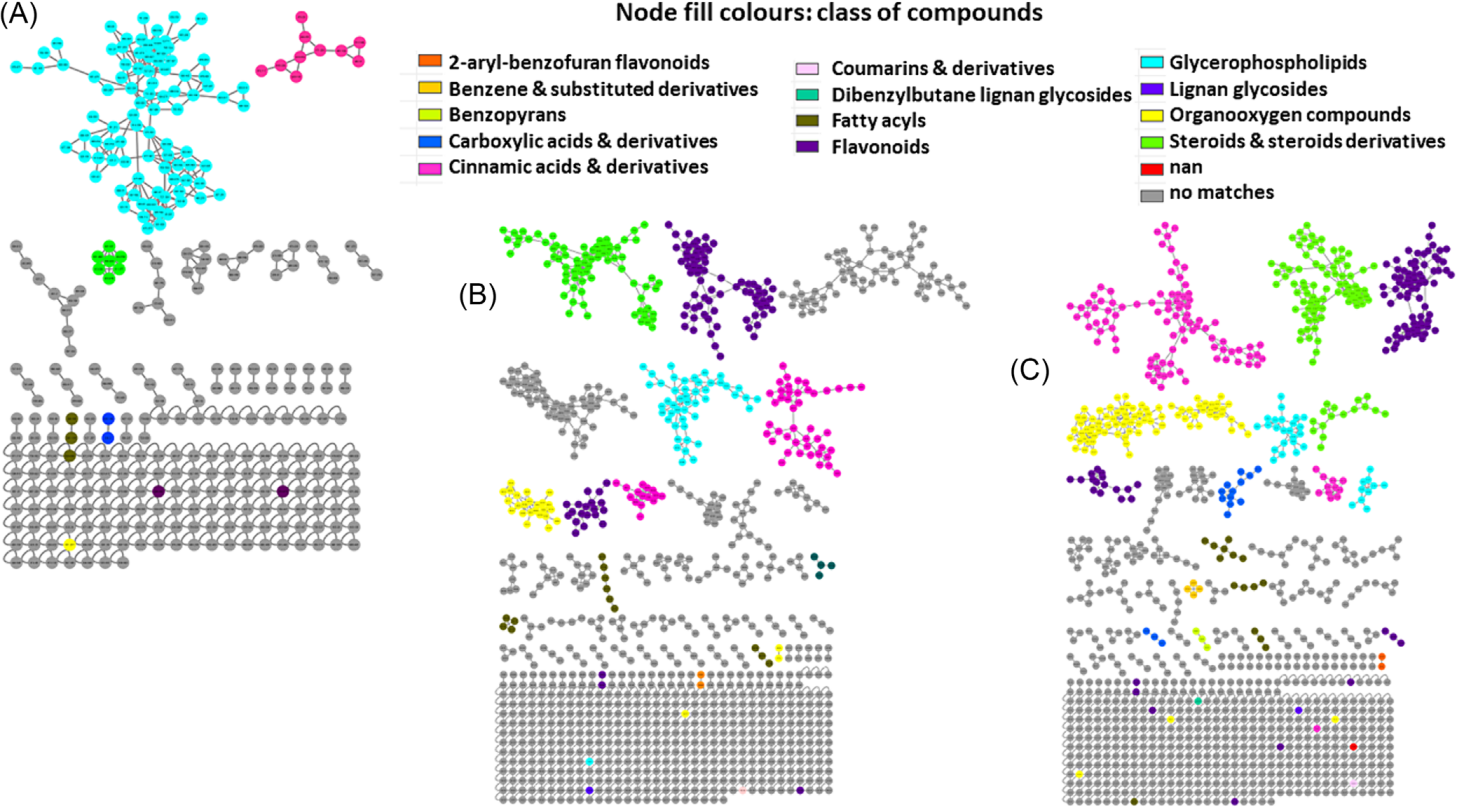
An enhanced molecular network of *Asparagus* species cladodes ethyl acetate (A), chloroform (B), and methanolic extracts analyzed by liquid chromatography–tandem mass spectrometry (LC–MS/MS) using electrospray ionization in negative mode, with flavonoids, cinnamic acids, glycerophospholipids, and organooxygen compounds as the major metabolite class identities.

To further explore the chemistries of the three species, UHPLC‐qTOF‐MS data were submitted to GNPS workflow for molecular network construction. MS‐based molecular networking connects mass spectra of molecules based on the similarity of their fragmentation patterns.^43^, ^44^ Molecular networking groups metabolites into molecular families based on their MS/MS fragmentation similarity.^45^ Figures 2 and S3–S5 show an enhanced classical molecular network generated with *in silico* tools housed in GNPS, namely, MolNetEnhancer, NAP, MS2LDA, and DEREPLICATOR. The enhanced network detected molecular families, subfamilies, and subtle structural differences among family members, increasing the reliability of the annotation.^46^, ^47^ Molecular networking allowed the annotation of various metabolites belonging to different chemical classes such as phenolics, glycerophospholipids, fatty acyls, organooxygen compounds, carboxylic acids, and benzenes. Furthermore, spectral library and manual inspection of MS/MS data allowed the annotation of 118 metabolites (illustrated by the sunburst in Figure 6 and Table S1).

The extraction of metabolites from the three *Asparagus* species cladodes with different solvents (Figure 2) indicated that the choice of solvent used for extraction has an impact on the efficiency of extraction. Topologically, it is evident that these solvents influenced the number of nodes generated in the networks. The network generated from ethyl acetate contained 327 nodes connected by 447 edges, with 57.80% of the nodes organized into 31 molecular clusters comprising two or more nodes each. In contrast, methanol resulted in 1098 nodes connected by 1626 edges, with 61.48% of the nodes organized into 87 molecular clusters. Chloroform yielded 1159 nodes connected by 1661 edges, with 62.81% of the nodes organized into 99 clusters. Notably, methanol and chloroform resulted in higher percentages of clustered nodes compared with ethyl acetate. As shown in Figure 2, the chloroform network displayed more clustered nodes and a greater number of propagated annotations.

The use of different solvents also influenced the chemical classification of metabolites. Ethyl acetate significantly limited the chemical classification, as many classes of extracted metabolites were excluded from the enhanced molecular network (Figure 2A). Despite excluding some classes, ethyl acetate was effective in extracting a large cluster of glycerophospholipids, indicating high structural diversity within this chemical class. Thus, ethyl acetate favored the extraction of non‐polar compounds. Conversely, methanol extracted a broader range of compound classes, including flavonoids, fatty acyls, organooxygen compounds, lignans, and benzenes, which were not recovered by ethyl acetate (Figure 2B). Additionally, the number of nodes and clusters for steroids and cinnamic acids was significantly higher in methanol compared with ethyl acetate.

The metabolome of *Asparagus* species extracted with chloroform was observed to include a larger class of metabolites, consisting of 2‐arylbenzofuran flavonoids, carboxylic acids, coumarins, and dibenzylbutane lignans, which were only seen when extracting with chloroform (Figure 2C). In addition to solvent comparisons, molecular networks for different solvents per species were generated, revealing distinct extraction profiles (Figures S3–S5). This indicates that the choice of solvent significantly impacts the overall metabolome by either excluding or emphasizing certain metabolite groups that are biologically important. Therefore, selecting the appropriate solvent for extracting and isolating bioactive compounds from plant materials is a critical factor in metabolomics studies.

**FIGURE 3:**
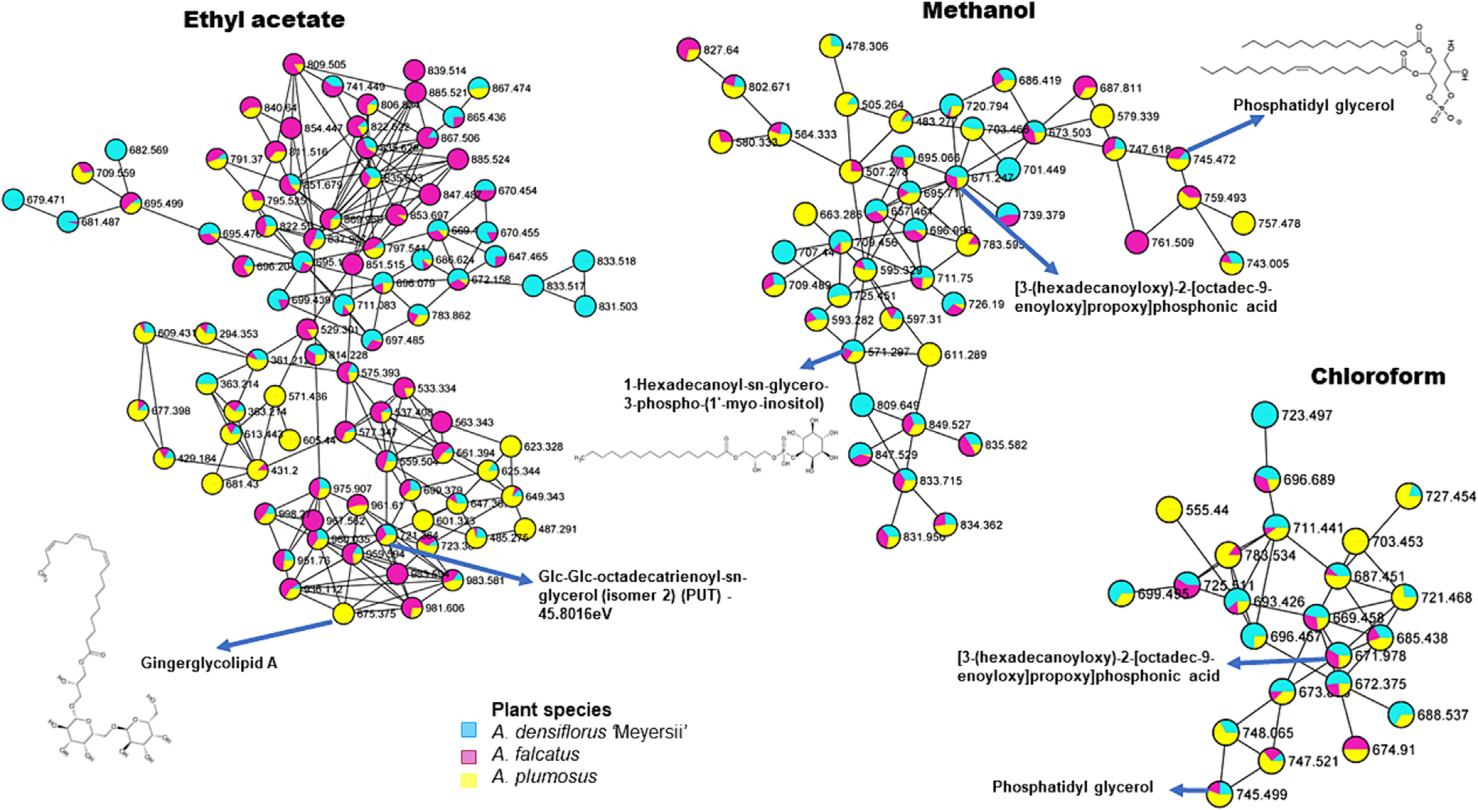
A zoomed‐in solvents molecular network of the glycerophospholipids class. Nodes from the selected molecular class are labeled with parent mass and displayed as pie charts that represent the distribution of the ion intensities of *A. densiflorus* ‘Meyersii’ (blue), *A. falcatus* (purple), and *A. plumosus* (yellow).

**FIGURE 4:**
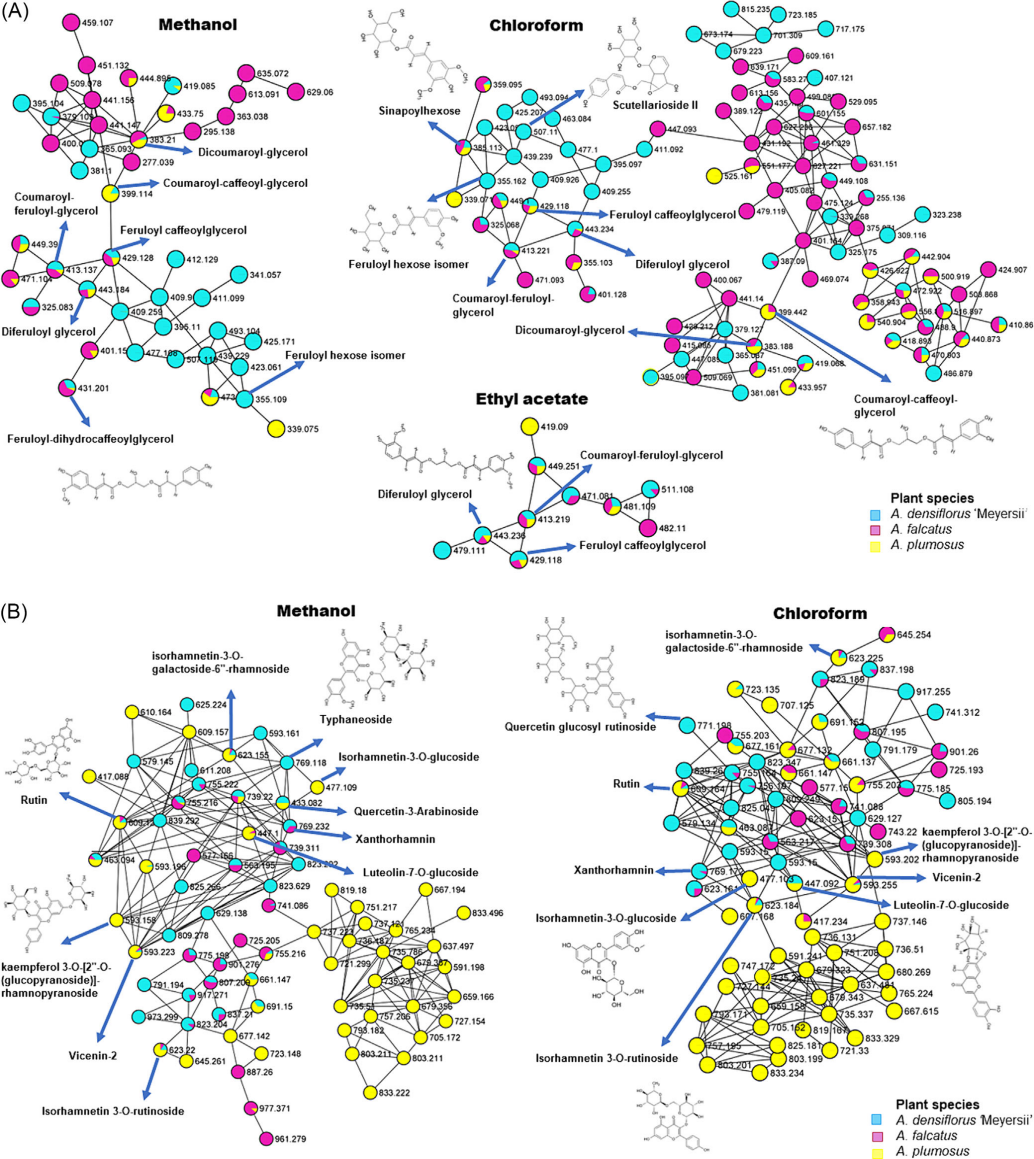
Zoomed‐in solvents molecular network of phenylpropanoids class (cinnamic acids [A] and flavonoids [B]). Nodes from the selected molecular class are labeled with parent mass and displayed as pie charts that represent the distribution of the ion intensities of *A. densiflorus* ‘Meyersii’ (blue), *A. falcatus* (purple), and *A. plumosus* (yellow).

**FIGURE 5:**
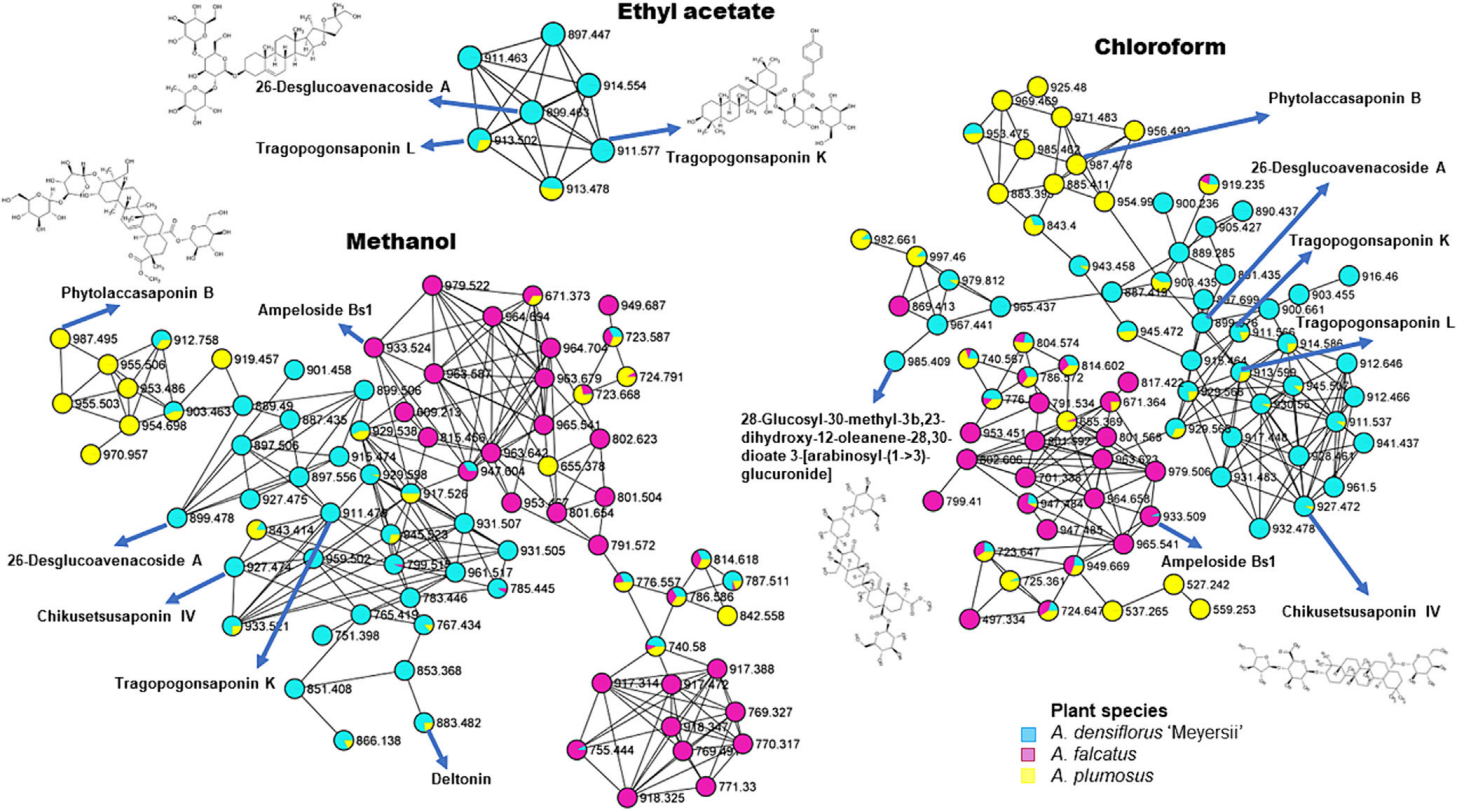
A zoomed‐in solvents molecular network of steroids class. Nodes from the selected molecular class are labeled with parent mass and displayed as pie charts that represent the distribution of the ion intensities of *A. densiflorus* ‘Meyersii’ (blue), *A. falcatus* (purple), and *A. plumosus* (yellow).

To further evaluate the influence of different solvents on molecular network topology and molecular families, the major class of compounds (cinnamic, steroids flavonoids, and glycerophospholipids) were examined in detail (Figures 3, 4, 5). This analysis aimed to explore the chemical diversity and compare the metabolome composition of the three *Asparagus* species cladodes. In the zoomed‐in networks, the analysis highlighted the differential distribution of glycerophospholipids when extracted with different solvents (Figure 3). Ethyl acetate yielded the highest number of glycerophospholipid nodes compared with the other solvents (ethyl acetate: 90 nodes, methanol: 47 nodes, chloroform: 23 nodes). Compounds within this class were distributed differently among the three solvents some metabolites were shared between all three solvents, others were shared between two solvents, and some were solvent specific. For example, gingerglycolipid A and Glc‐Glc‐octadecatrienoyl‐sn‐glycerol were only present in extracts obtained with ethyl acetate, while 1‐hexadecanoyl‐sn‐glycero‐3‐phospho‐(1′‐myo‐inositol) was found only in methanol extracts. Additionally, methanol and chloroform allowed the extraction of similar compounds, such as phosphotiglycerol and [3‐(hexadecanoyloxy)‐2‐[octadec‐9‐enoyloxy]propoxy] phosphonic acid. At the species level, similar observations were made, with some metabolites being common across all three species, common to two species or species‐specific (Figure 3).

Considering the molecular family of phenylpropanoids (Figure 4), the use of different solvents was observed to affect the metabolome of this chemical class. Significant variation in the number of nodes among the three solvents was noted in the cinnamic acids class: ethyl acetate had 10 nodes, methanol had 44 nodes, and chloroform had 92 nodes (Figure 4A). Chloroform extracted a larger cluster of cinnamic acids; for instance, some compounds annotated were not present when extracted with methanol or ethyl acetate. As observed in Figure 4A, scutellarioside II and sinapoylhexose were extracted only with chloroform.

Regarding species comparison within this class, fewer compounds were shared between nodes and most were unique (e.g., scutellarioside II and feruloylhexose isomers were only found in *A. densiflorus*). In comparison with flavonoids (Figure 4B), there was a slight variation in the number of nodes between methanol and chloroform extracts (ethyl acetate was absent, with methanol having 74 nodes and chloroform having 75 nodes). Certain metabolites discriminated between the two solvents; for example, typhaneoside was present only in methanol extracts, while xanthorhamnin was extracted with chloroform. In contrast, steroids also showed variation in terms of number of nodes among the three solvents; ethyl acetate consisted of seven nodes while methanol having 77 and chloroform consisted of 81 nodes; thus, chloroform and methanol extracted steroids with slight variation in terms of the number of nodes; however, chloroform has more nodes (Figure 5). Metabolite at *m/z* 985, annotated as 28‐glucosyl‐30‐methyl‐3b,23‐dihydroxy‐12‐oleanene‐28,30‐dioate‐3‐[arabinosyl(1>3) glucuronide], falls among the compounds which were extracted only by chloroform and not the other two solvents. Furthermore, these solvents also revealed more unique compounds within the three species. For example, metabolite at *m/z* 933, which was annotated as ampeloside Bs1, was only present in *A. falcatus* and was only extracted by methanol.

#### 3.2.1 Chemotaxonomic comparison of *Asparagus* species

**FIGURE 6:**
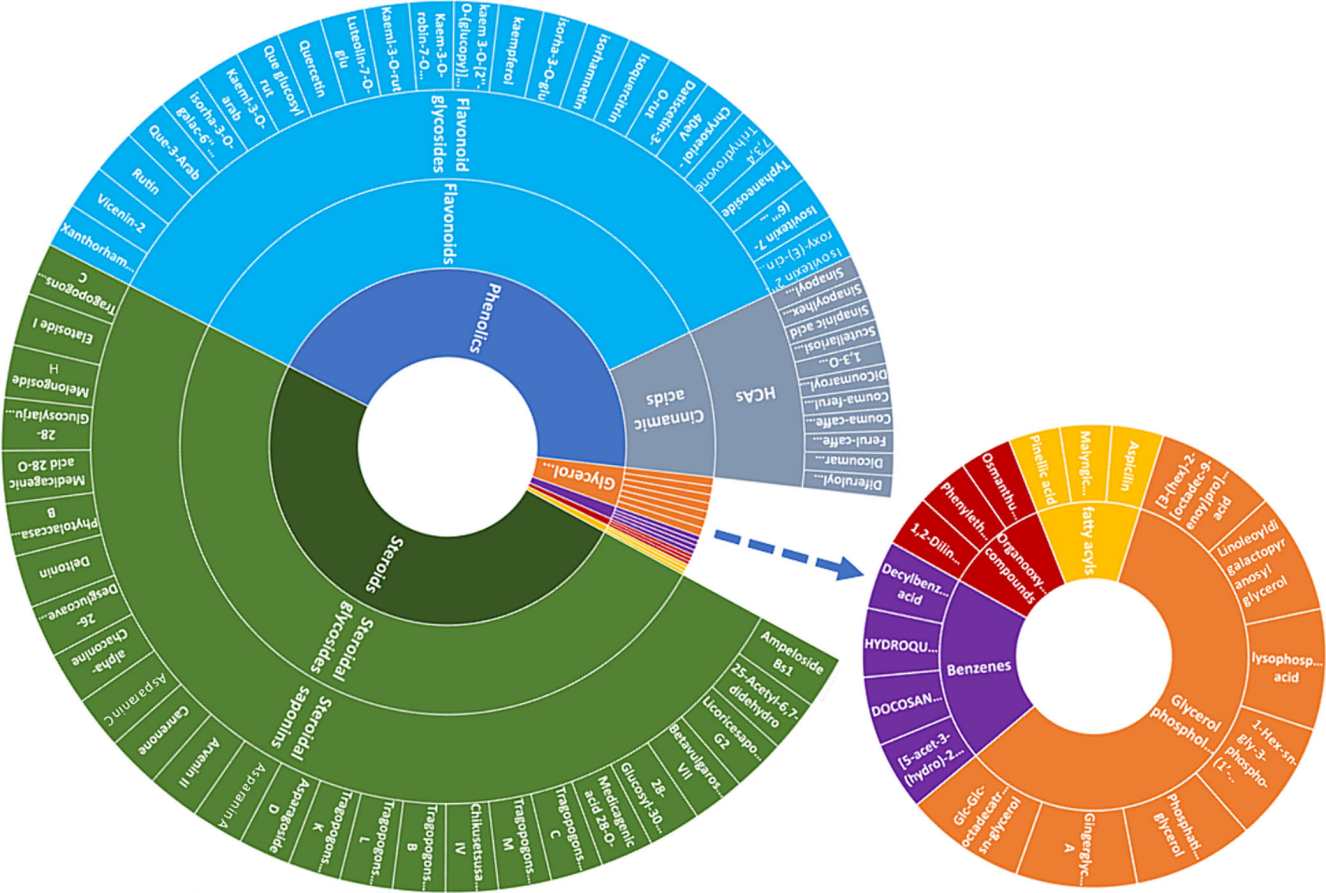
Sunburst plot representing the classification of the annotated metabolites. The class of annotated metabolites include phenolics, steroids, organooxygen compounds, benzenes, and fatty acyls.

*Asparagus* is known to possess chemo‐diverse health‐promoting compound classes such as triterpenoids, saponin glycosides, hydroxycinnamic acids flavonoids, phenolics, and alkaloids.^1^, ^48^ The results of this study revealed the main class of annotated metabolites as flavonoids, cinnamic acids, steroidal saponins, organooxygen compounds, benzenes, and fatty acyls. Among these, steroidal saponins and phenolics (including cinnamic acids and flavonoids) were identified as the most dominant classes of compounds across the three *Asparagus* species (Figure 6).

**FIGURE 7:**
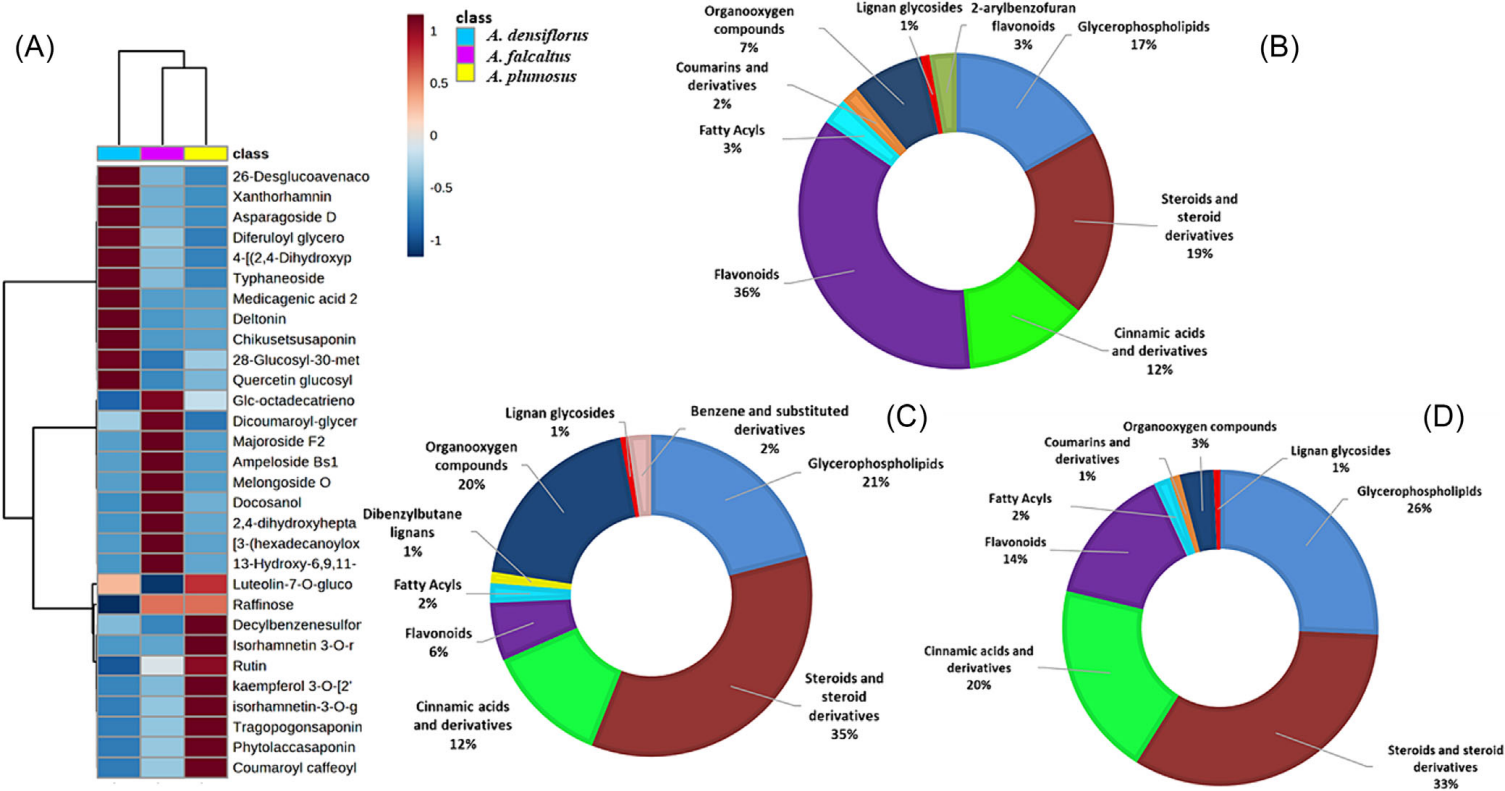
Metabolite distribution in *Asparagus* species. (A) A clustered averaged heatmap with a dendrogram showing hierarchical clustering on compound distributions. The color code on top indicates the species to which a column belongs: *A. densiflorus* ‘Meyersii’ (blue), *A. falcatus* (purple), and *A. plumosus* (yellow). (B–D) Distribution of compound classes in *A. plumosus* (B), *A. falcatus* (C), and *A. densiflorus* ‘Meyersii’ (D). Numbers within the pie charts indicate the total number of compounds of the class annotated in this species.

These metabolomics insights paved the way for exploring the distribution of compound classes among the three species. Differences and common features between the species were observed. Figure 7 summarizes the distribution of classes among the three species, with *A. densiflorus* dominating in glycerophospholipids and steroids (Figure 7D), *A. falcatus* in steroids (Figure 7C), and *A. plumosus* in flavonoids (Figure 7B). A lack of benzenes in *A. densiflorus* and *A. plumosus* (Figure 7B,D) was also noted. To investigate variations among the identified metabolites, a hierarchical clustering heatmap analysis (Figure 7A) was performed using MetaboAnalyst. The heatmaps revealed distinct diversities between solvent extracts of *Asparagus* species cladodes in terms of metabolite abundance. The saponins class was highly abundant in *A. densiflorus*, while many flavonoids were more abundant in *A. plumosus* **(**Figure 7A). In summary, the metabolomics insights indicate that each *Asparagus* species has a unique metabolite profile, which can help in distinguishing between them based on the abundance and distribution of various compound classes.

**FIGURE 8:**
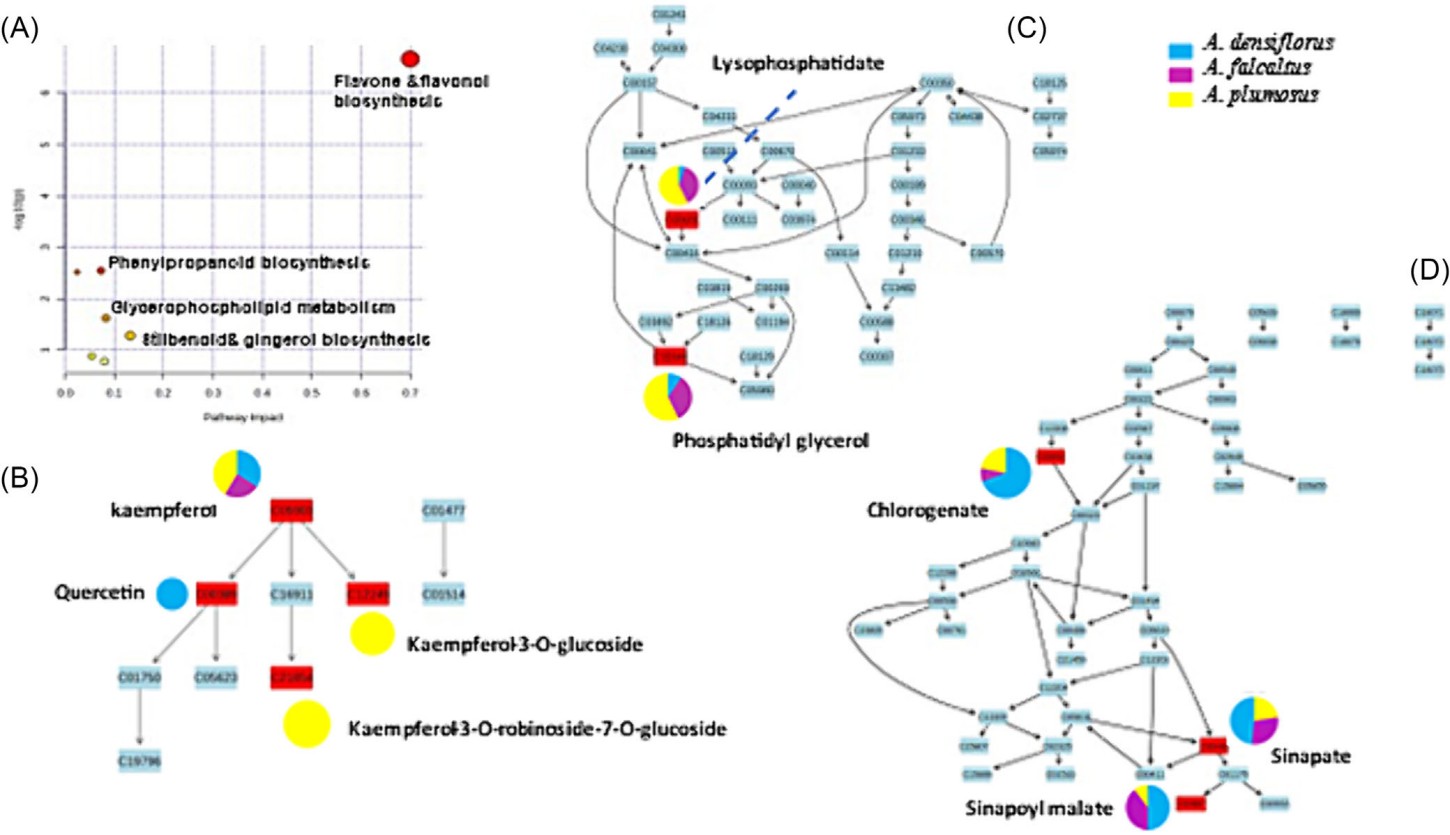
MetaboAnalyst pathway analysis. Metabolism pathways identified in *Asparagus* species (A). An indication of the flavone and flavonol biosynthesis pathway (B), glycerophospholipid pathway (C), and phenylpropanoid biosynthesis pathway (D). The abundance of each metabolite in three distinct species of *Asparagus* is depicted in pie charts.

Furthermore, the annotated metabolites listed in Table S1 and illustrated in the sunburst chart in Figure 6 were used to perform metabolic pathway analysis in MetaboAnalyst. The pathways are arranged according to pathway impact values (*x* axis), which indicate pathway topology analysis and *p* values (*y* axis), which indicate pathway enrichment analysis. The relative intensity of different metabolites within the three species is shown in the pie charts. The analysis revealed several impactful metabolic pathways, including the glycerophospholipid pathway, flavonoid biosynthesis pathway, flavone and flavonol biosynthesis pathway, lysine biosynthesis pathway, galactose metabolism pathway, glycerolipid metabolism pathway, phenylpropanoid biosynthesis pathway, and stilbenoid biosynthetic pathway (Figure 8).

From a chemotaxonomic perspective, these findings highlight distinctive metabolic profiles among the *Asparagus* species. The flavone and flavonol biosynthesis pathway was identified as the most statistically significant, followed by the flavonoid biosynthesis, phenylpropanoid biosynthesis, and glycerophospholipid metabolism pathways (as shown in Figure 8A). In the flavone and flavonol biosynthesis pathway, quercetin, kaempferol, kaempferol‐3‐*O*‐rhamnoside‐7‐*O*‐rhamnoside, and kaempferol‐3‐*O*‐glucoside were mapped and were more abundant in *A. plumosus* (Figure 8B). The glycerophospholipid biosynthesis pathway revealed the mapping of phosphatidylglycerol and lysophosphatidate, which were present across all three species with varying relative concentrations (mostly abundant in *A. plumosus*) (Figure 8C). In the enriched phenylpropanoid biosynthesis pathway, three hydroxycinnamates (chlorogenate, sinapoyl malate, and sinapate) were mapped and were present across all three species, with the highest abundance in *A. densiflorus* (Figure 8D).

These chemotaxonomic insights suggest that LC–MS‐based metabolomics and molecular networking, with the appropriate choice of extraction solvent, can effectively differentiate among *Asparagus* species based on their metabolite profiles. This approach enhances our biological understanding of *Asparagus* species cladodes and supports the development of a more refined taxonomic classification based on metabolic characteristics.

## 4 DISCUSSIONS

The use of medicinal plants as the source of bioactive compounds for the treatment of various alignments has increasingly gained a considerable attention. The current study used *Asparagus* as the main natural source of phytochemical compounds. *Asparagus* species are herbal plants that are well documented in literature containing various health‐promoting compounds from diverse classes such triterpenoids, saponin glycosides, hydroxycinnamic acids, and flavonoids.^1^, ^11^ In this study, LC–MS‐based metabolomics and molecular networking was used to compare metabolites profiles of different solvent extracts of *Asparagus* species cladodes. Cladodes were chosen for metabolite analysis due to their high concentration of bioactive compounds compared with other plant parts, such as roots or whole plants. Additionally, harvesting cladodes causes minimal harm to the plant, allowing for sustainable extraction.^49^, ^50^, ^51^, ^52^, ^53^

In metabolomics studies, extraction is the most critical step for recovering and isolating bioactive compounds from plant materials. The efficacy of extraction depends on various factors, such as temperature, time, pH, the composition of phytochemicals, and the solvent used.^29^, ^54^ Therefore, this study recognized the solvent as one of the most critical parameters affecting the efficiency of extraction and the metabolite composition obtained. The current study used methanol, ethanol, and chloroform to extract phytochemical compounds from *A. densiflorus* ‘Meyersii’, *A. falcatus*, and *A. plumosus*. Using PCA, the results showed that the identified metabolites varied among the three species and that different solvents resulted in different sample clustering, indicating significant metabolic differences (Figure 1). The polarity of the solvent used impacts extraction efficiency, which may explain the wide variation in the composition of phytochemical compounds in the extracts.^29^, ^55^ Interestingly, methanol and chloroform were revealed to cluster at the same point which suggests that the two solvents were closely related in terms of metabolite composition they are extracting (Figure 1E).

Molecular networking was then applied to explore the chemical diversity within the three solvent extracts of *Asparagus* species, thereby outlining the similarities and differences. Ethyl acetate appeared to be a less effective solvent for extraction but favored non‐polar compounds such as glycerophospholipids (Figure 2A). This is because ethyl acetate is considered a moderately polar (semi‐polar) organic solvent, which can recover available lipids from sample extracts.^56^ In contrast, higher extraction efficiencies were observed with methanol and chloroform, indicating that these solvents were more effective (Figure 2B,C). Methanol extracted a broader range of compound classes, some of which were not recovered by ethyl acetate (e.g., flavonoids, fatty acyls, organooxygen compounds, lignans, and benzenes). However, the metabolome of *Asparagus* species extracted with chloroform contained a larger variety of metabolites, including some that were unique to this solvent. This may be due to the high levels of lipids and lipid‐like molecules (such as steroids, glycerophospholipids, and fatty acids) present in *Asparagus* species. Additionally, when chloroform extracts were returned to a liquid state, methanol was used and pairing polar solvents with non‐polar organic solvents like chloroform which is volatile, non‐reactive, and immiscible with water facilitates the separation of both polar and non‐polar metabolites.^23^, ^25^ Terpenoids, steroids, and glycerophospholipids, known for their low polarity, dissolve well in lipophilic solvents such as chloroform.^48^, ^57^ Conversely, highly polar compounds like polyphenols, carbohydrates, and amino acids dissolve in methanol.^58^ Consistent with the findings of this study, both methanol and chloroform enable comprehensive profiling of plant metabolites, capturing both polar and non‐polar compounds.

In accordance with chemotaxonomic classification of *Asparagus*, the content of bioactive compounds was identified as the following class: phenolics (flavonoids and HCAs), glycerophospholipids, steroids, organooxygen compounds benzenes, and fatty acyls (Figure 6 and Table S1). However, steroids and phenolics were the most dominating class of annotated compounds. Studies have demonstrated steroids from *Asparagus* possess powerful anti‐inflammatory, anticancers, and antimalarial activities.^1^, ^59^ Flavonoids are the most diverse class of plant secondary metabolites, and they are associated with various health benefits because of their free radical‐scavenging properties.^60^, ^61^ Moreover, flavonoids of these species (specifically quercetin, isorhamnetin, and kaempferol) have been found to have blood pressure‐lowering, anti‐inflammatory, antiviral, and anticancer effects in humans.^62^ This suggests that these *Asparagus* species may be helpful as disinfectants and in treating conditions linked to oxidative stress; phenolic compounds have been used extensively to prevent the growth of microorganisms.^63^, ^64^ This could support the traditional uses of *Asparagus* in medicine to treat kidney stones, skin conditions, blood dysentery, urinary disorders, and diarrhea. Steroidal saponins also help to fight microorganisms that are resistant to antibiotics such as yeast and other fungi.^65^, ^66^ Studies have demonstrated that steroids from *Asparagus* possess powerful anti‐inflammatory,^59^, ^67^ anticancer,^68^ and antimalarial activities.^69^, ^70^ The highest levels of phenolics were observed in *A. plumosus*, glycerophospholipids and steroids were more abundant in *A. densiflorus*, while *A. falcatus* had more organooxygen compounds and steroids (Figure 7).

To investigate the variations among the identified metabolites, a hierarchical clustering heatmap analysis was performed. The heatmaps revealed unique diversities between solvents extract of *Asparagus* species terms of metabolite abundance. Specifically, the class of saponins was highly abundant in *A. densiflorus*, while many flavonoids were more abundant in *A. plumosus* (Figure 7A). To further explore the chemistry of the three species, pathway analyses were also conducted. The most statistically significant pathways identified were the glycerophospholipid pathway, flavone, and flavonol biosynthesis pathways, phenylpropanoid biosynthesis pathway, and stilbenoid biosynthetic pathways (Figure 8). Under abiotic stress circumstances, the phenylpropanoid metabolic pathway is activated, leading to the buildup of several phenolic compounds that have the ability to scavenge harmful reactive oxygen species.^71^, ^72^ The glycerophospholipid pathway's metabolites are essential for preserving the integrity of cell membranes against hypoxic stress and preventing cell damage.^73^, ^74^ From a chemotaxonomic perspective, *A. densiflorus*, *A. falcatus*, and *A. plumosus* all contain saponins, glycerophospholipids, and flavonoids, reinforcing their classification within the *Asparagaceae* family. The presence of these compound classes across the species supports their taxonomic grouping. However, the types and concentrations of these compounds differ among the species, reflecting their unique properties and applications. *A. densiflorus* and *A. falcatus* are notable for their additional therapeutic properties, while *A. plumosus* is more associated with general health benefits. Therefore, these findings suggest that the right choice of solvent used for extraction could be used for maximizing the identification phytochemical compounds with more practical value. Additionally, the chemotaxonomic comparison highlights how the chemical constituents of these *Asparagus* species not only define their classification but also underscore their varied medicinal and practical uses.

## 5 CONCLUSIONS

This study provided a comprehensive analysis of metabolites in different solvent extracts from *Asparagus* species cladodes. The results suggest a dynamic range of metabolites across the three *Asparagus* species, depending on the solvent used for extraction. LC–MS‐based metabolomics and molecular networking were employed to detect variations between solvent extracts of *Asparagus* cladodes. A total of 118 metabolites were annotated from the three different solvents, with chloroform and methanol extracts identified as containing more metabolites. Although ethyl acetate was a less effective solvent overall, it enabled the extraction of a significant amount of glycerophospholipids. Additionally, chloroform extracts contained a broader range of compound classes. This study underscores molecular networking as a highly valuable tool in metabolomics, as it delineated similarities and differences among different extracts, even when they clustered closely on PCA plots. These findings suggest that LC–MS‐based metabolomics and molecular networking, when complemented by the appropriate choice of solvent for extraction, can maximize the identification of important metabolites with potential significance for human health and can be utilized in future studies of these species.

## Supporting information


**Figure S1:** Seedling pictures of *A. densiflorus ‘*Meyersii’ [A], *A. falcatus* [B], and *A. plumosus* [C].
**Figure S2:** Representative LC–MS chromatograms of methanolic, chloroform and ethyl acetate extracts of *A. densiflorus.* The pink overlays on the chromatograms represent the MS2 (ions from the MS1 spectra are subjected to collision energy, and then selectively fragmented).
**Figure S3:** PCA score plots for the discrimination between different solvent extracts of *Asparagus* species. Each spot represents one sample of methanolic extracts (*A. densiflorus ‘*Meyersii’ [blue], *A. falcatus* [yellow], and *A. plumosus* [light green]), ethyl acetate ( *A. densiflorus* [green], *A. falcatus* [pink], and *A. plumosus* [orange]), and chloroform extracts ( *A. densiflorus* ‘Meyersii’ [red], *A. falcatus* [light blue], and *A. plumosus* [purple]). The ellipses show the differences and similarities between the groups. The model obtained was a two‐dimensional component model that explains 37.9% variation.
**Figure S4:** An enhanced molecular network of *A. densiflorus* extracted by ethyl acetate, chloroform, and methanolic extracts and analysed by LC–MS/MS using electrospray ionisation in negative mode.
**Figure S5:** An enhanced molecular network of *A. falcatus* extracted by ethyl acetate, chloroform, and methanolic extracts and analysed by LC–MS/MS using electrospray ionisation in negative mode.
**Figure S6:** An enhanced molecular network of *A. plumosus* extracted by ethyl acetate, chloroform, and methanolic extracts and analysed by LC–MS/MS using electrospray ionisation in negative mode.
**Table S1:** Summary of annotated metabolites of different solvent extracts of *Asparagus* species.

## Data Availability

The original contributions presented in the study are included in the article/Supporting Information. Further inquiries can be directed to the corresponding author.
