# Supplementary material for: Comparative metabolites profiling of different solvent extracts of Asparagus species cladodes using liquid chromatography–mass spectrometry‐based metabolomics and molecular networking
Source: Phytochem Anal. 2024 Sep 9;36(3):506–19. doi: 10.1002/pca.3446 (PMC11986901; doi:10.1002/pca.3446)
Supplement: Supplementary file 1 — Figure S1: Seedling pictures of A. densiflorus ‘Meyersii’ [A], A. falcatus [B], and A. plumosus [C]. Figure S2: Representative LC–MS chromatograms of methanolic, chloroform and ethyl acetate extracts of A. densiflorus. The pink overlays on the chromatograms represent the MS2 (ions from the MS1 spectra are subjected to collision energy, and then selectively fragmented). Figure S3: PCA score plots for the discrimination between different solvent extracts of Asparagus species. Each spot represents one sample of methanolic extracts ( A. densiflorus ‘Meyersii’ [blue], A. falcatus [yellow], and A. plumosus [light green]), ethyl acetate ( A. densiflorus [green], A. falcatus [pink], and A. plumosus [orange]), and chloroform extracts ( A. densiflorus ‘Meyersii’ [red], A. falcatus [light blue], and A. plumosus [purple]). The ellipses show the differences and similarities between the groups. The model obtained was a two‐dimensional component model that explains 37.9% variation. Figure S4: An enhanced molecular network of A. densiflorus extracted by ethyl acetate, chloroform, and methanolic extracts and analysed by LC–MS/MS using electrospray ionisation in negative mode. Figure S5: An enhanced molecular network of A. falcatus extracted by ethyl acetate, chloroform, and methanolic extracts and analysed by LC–MS/MS using electrospray ionisation in negative mode. Figure S6: An enhanced molecular network of A. plumosus extracted by ethyl acetate, chloroform, and methanolic extracts and analysed by LC–MS/MS using electrospray ionisation in negative mode. Table S1: Summary of annotated metabolites of different solvent extracts of Asparagus species. [file PCA-36-506-s001.docx]

**Comparative** **metabolites profiling of different solvent extracts of *Asparagus* species through** **LC-MS-based metabolomics and molecular networking**

**Pfano Witness Maphari ^1^, Mthokozisi B. C. Simelane^1^, Ntakadzeni Edwin Madala ^2^, and Msizi Innocent Mhlongo ^1*^**

^1^Department of Biochemistry, Faculty of Science, University of Johannesburg, Auckland Park, 2006, Gauteng, South Africa.

^2^Department of Biochemistry and Microbiology, Faculty of Science, Engineering and Agriculture, University of Venda, Private Bag X5050, Thohoyandou, 0950, Limpopo, South Africa.

*Correspondence: E-mail: [mmhlongo@uj.ac.za](mailto:mmhlongo@uj.ac.za); Tel.: +27-11-559-4573

**Supplements Files**

**
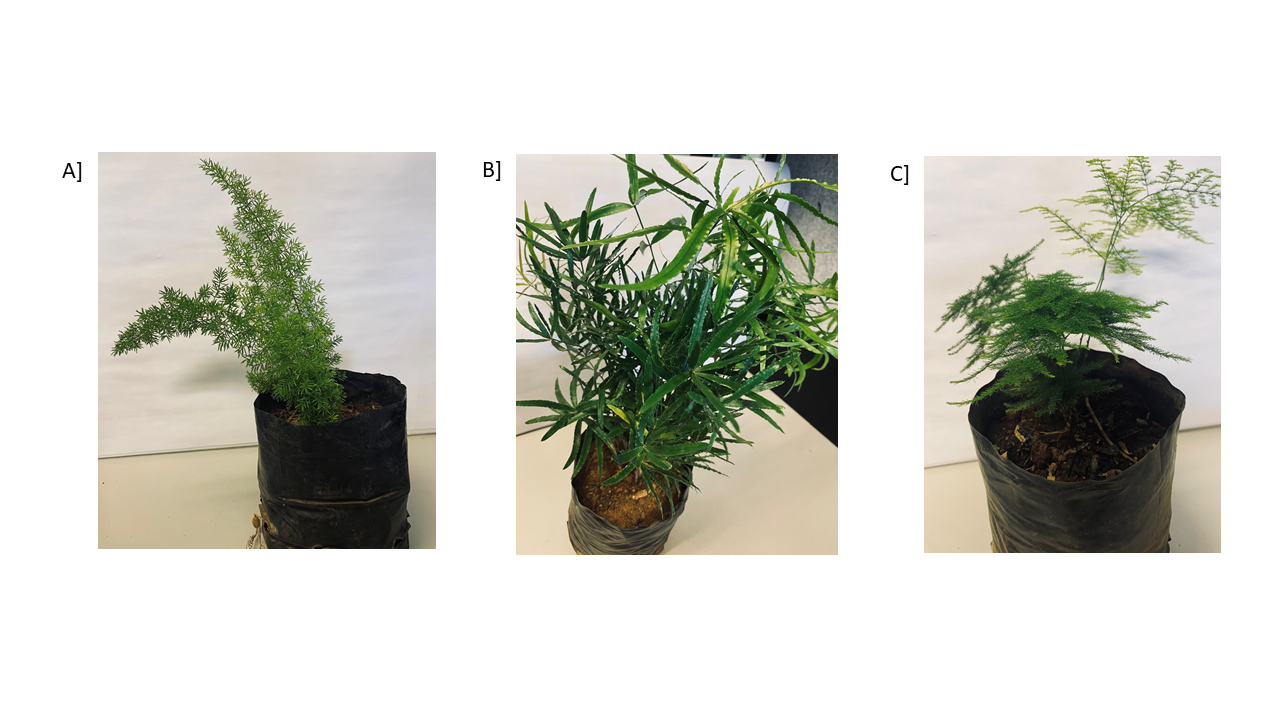
**

**Figure S1:** Seedling pictures of *A.* *densiflorus ‘*Meyersii’ [A], *A. falcatus* [B], and *A. plumosus* [C].

**
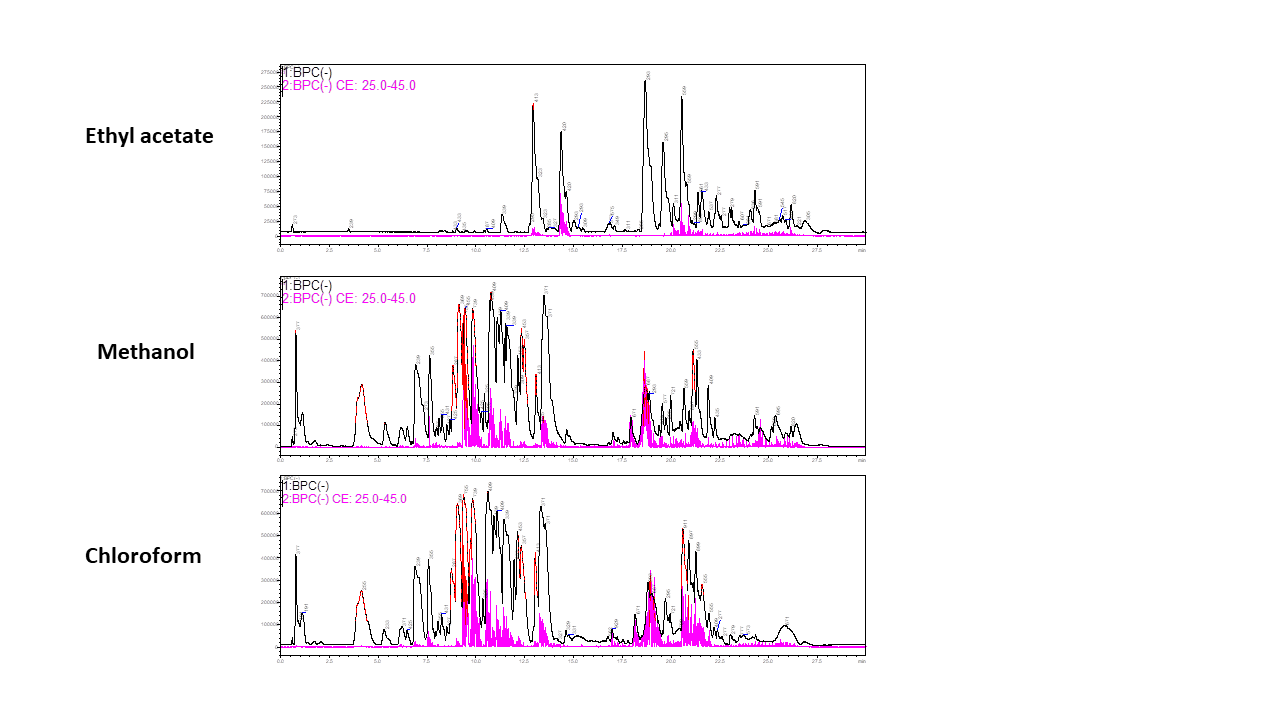
**

**Figure S2:** Representative LC-MS chromatograms of methanolic, chloroform and ethyl acetate extracts of

*A. densiflorus.* The pink overlays on the chromatograms represent the MS2 (ions from the MS1 spectra are subjected to collision energy, and then selectively fragmented)


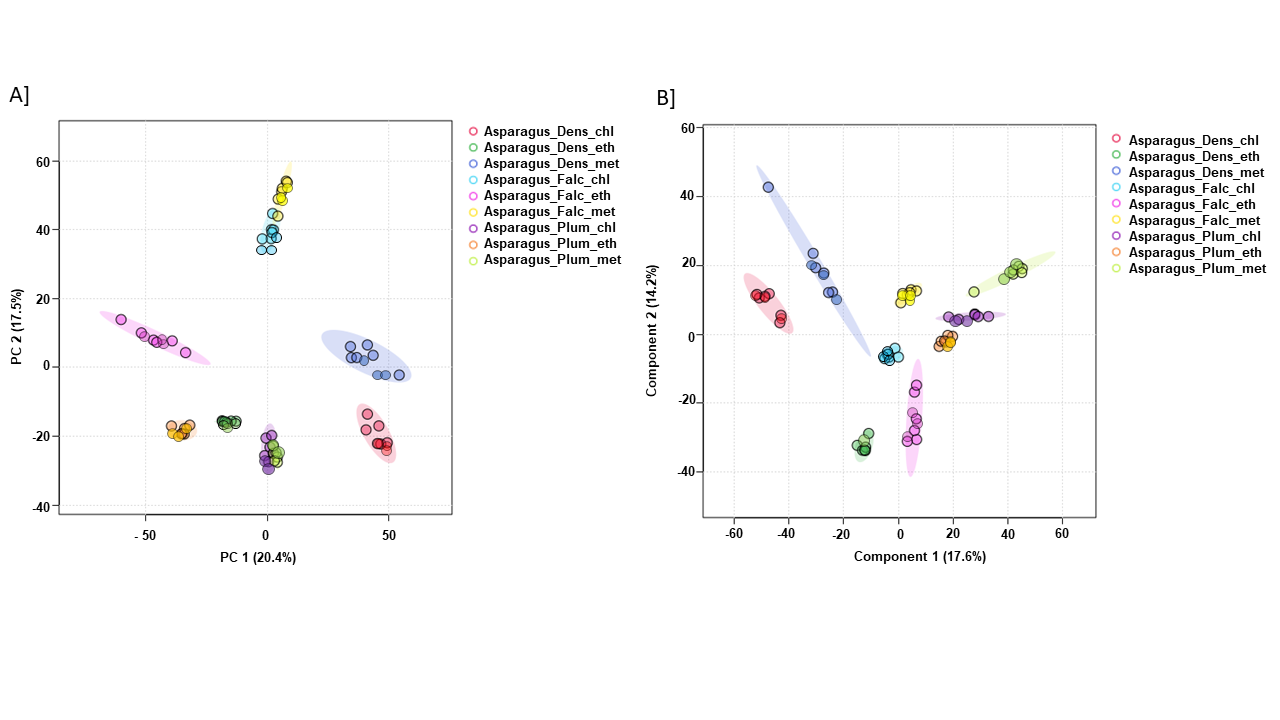


**Figure S3:** PCA score plots for the discrimination between different solvent extracts of *Asparagus* species. Each spot represents one sample of methanolic extracts (*A.* *densiflorus ‘*Meyersii’ [blue], *A. falcatus* [yellow], and *A. plumosus* [light green]), ethyl acetate (*A. densiflorus* [green], *A. falcatus* [pink], and *A. plumosus* [orange]), and chloroform extracts (*A. densiflorus* ‘Meyersii’ [red], *A. falcatus* [light blue], and *A. plumosus* [purple]). The ellipses show the differences and similarities between the groups. The model obtained was a two-dimensional component model that explains 37.9% variation.


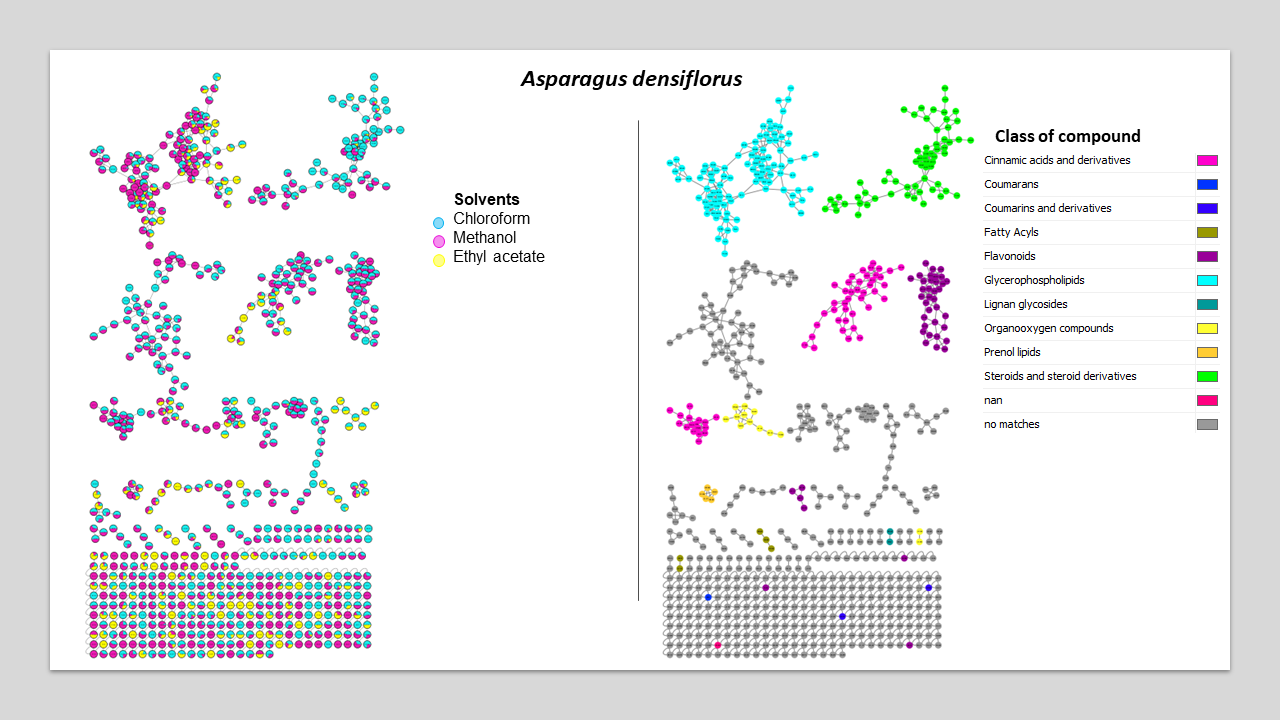


**Figure S4:** An enhanced molecular network of *A. densiflorus* extracted by ethyl acetate, chloroform, and methanolic extracts and analysed by LC-MS/MS using electrospray ionisation in negative mode.


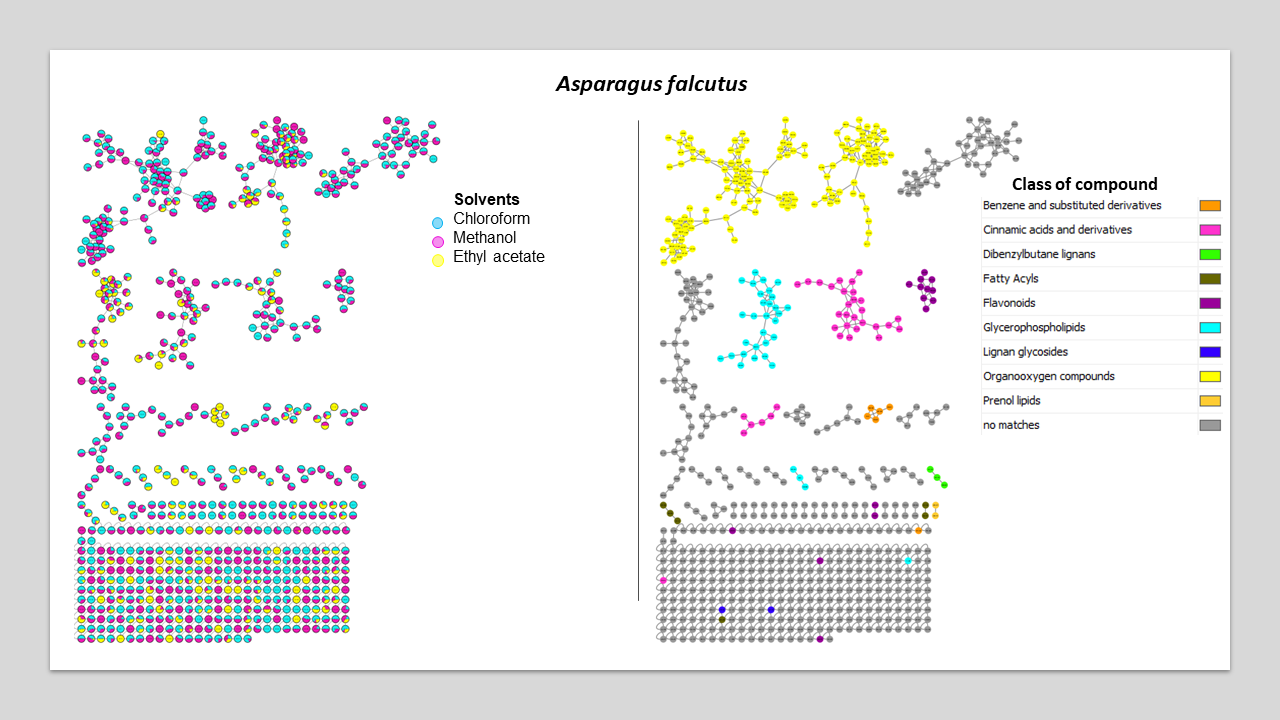


**Figure S5:** An enhanced molecular network of *A. falcatus* extracted by ethyl acetate, chloroform, and methanolic extracts and analysed by LC-MS/MS using electrospray ionisation in negative mode.


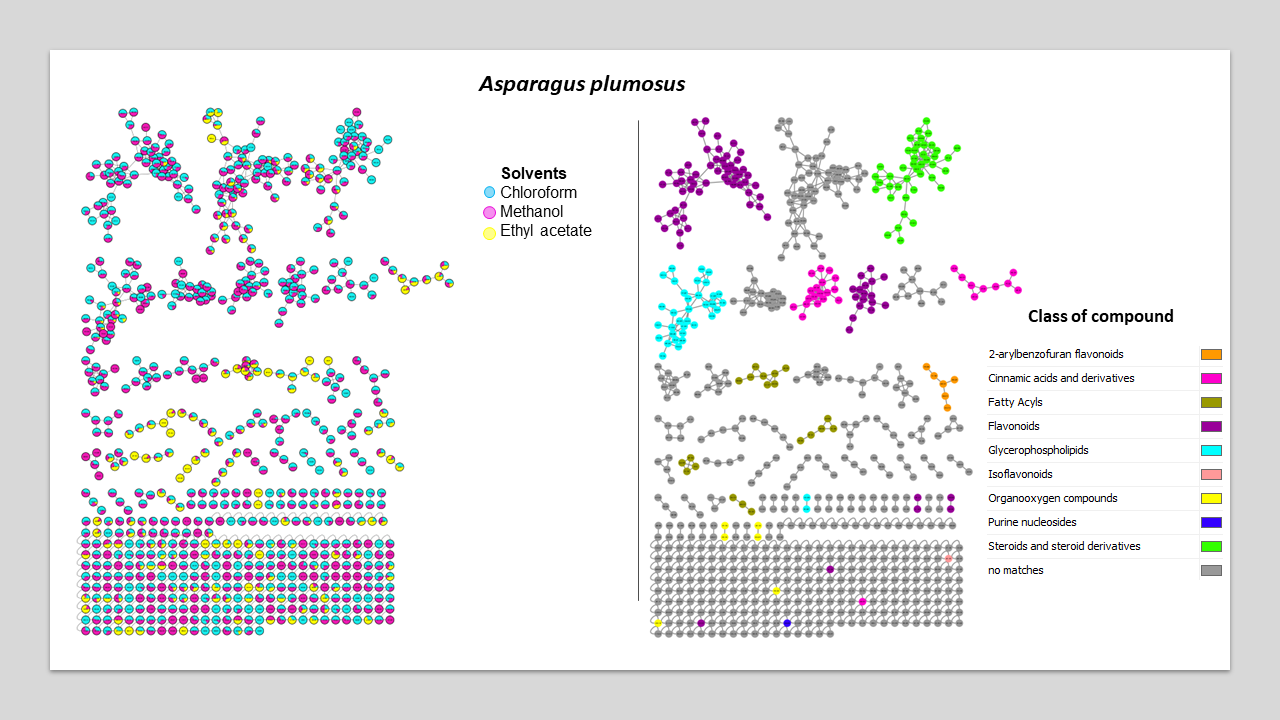


**Figure S6:** An enhanced molecular network of *A. plumosus* extracted by ethyl acetate, chloroform, and methanolic extracts and analysed by LC-MS/MS using electrospray ionisation in negative mode.

**Table S1:** Summary of annotated metabolites of different solvent extracts of *Asparagus* species

| **m/z (M-H)** | **rt min** | **Formula** | **Compound name** | **Fragment ions** | **A. f _et^1^** | **A. p _et^2^** | **A. d _et^3^** | **A. f _met^4^** | **A. p _met^5^** | **A. d**  **_met^6^** | **A. f**  **_Chl^7^** | **A. p**  **_chl^8^** | **A. d**  **_chl^9^** |
| --- | --- | --- | --- | --- | --- | --- | --- | --- | --- | --- | --- | --- | --- |
|  |  |  | **Cinnamic acids** |  |  |  |  |  |  |  |  |  |  |
| 443.176 | 8.90 | C23H24O9 | Diferuloyl glycerol (Glyceryl 1,2-diferulate) | 249.36, 193.10, 134.03 | * | * | * | * | * | * | * | * | * |
| 383.11 | 8.49 | C21H20O7 | Dicoumaroyl glycerol | 219.14, 163.06, 119.03 |  |  |  | * | * | * | * | * | * |
| 429.120 | 8.01 | C22H22O9 | Feruloyl caffeoylglycerol | 193.10, 179.03, 134.03 | * | * | * | * | * | * | * | * | * |
| 399. 122 | 7.93 | C21H20O8 | Coumaroyl-caffeoyl-glycerol | 253.23, 163.06, 179.03, 161.05, 135.03 |  |  |  | * | * | * | * | * | * |
| 413. 187 | 9.04 | C22H22O8 | Coumaroyl-feruloyl-glycerol | 235.03,173.02, 161.05, 135.03 | * | * | * | * | * | * | * | * | * |
| 483.143 | 6.58 | C21H20O7 | DiCoumaroyl-glycerol | 163.06, 119.03, 219.07 |  |  |  | * | * | * | * | * | * |
| 431.243 | 11.18 | C22H24O9 | 1,3-O-Feruloyl-dihydrocaffeoylglycerol | 295.06, 236.45, 235.03, 193.10, 160.08, 137.05 |  |  |  | * | * | * |  |  |  |
| 507.295 | 10.93 | C24H28O12 | Scutellarioside II | 373.63, 313.10 |  |  |  |  |  |  |  |  | * |
| 223.056 | 6.63 | C11H12O5 | Sinapinic acid | 205.05 |  |  |  | * | * | * | * | * | * |
| 385.114 | 6.23 | C17H22O10 | Sinapoylhexoside (isomer of 955) -(1-o-Sinapoylglucose) | 223.08, 205.05 |  |  |  |  |  | * |  |  | * |
| 339.071 | 6.66 | C15H16O9 | Sinapoyl malate | 223.08,164.25, 149.12, 121.03 |  |  |  | * | * | * | * | * | * |
| 929.269 | 8.33 | C44H50O22 | 2-Feruloyl-1,2'-disinapoylgentiobiose | 913.68, 485.23, 195.04, 193.10, 137.05 |  |  |  |  |  |  |  |  | * |
| 515.119 | 7.69 | C25H24O12 | 3,4-Di-O-caffeoylquinic acid | 492.19, 353.23, 335.33, 173.03 |  |  |  |  |  |  | * | * | * |
| 353.311 | 6.48 | C16H18O9 | Chlorogenic Acid | 191.10 |  |  |  | * | * | * | * | * | * |
| 473.289 | 10.90 | C22H18O12 | Dicaffeoyltartaric acid | 295.08, 179.03, 161.05, 135.03 |  |  |  |  |  |  | * | * | * |
| 179.034 | 7.18 | C9H8O4 | Caffeic acid | 163.06,1 35.03 |  |  |  | * | * | * | * | * | * |
| 193.103 | 8.34 | C10H10O4 | Ferulic acid | 178.08, 149.04, 134.04 |  |  |  | * | * | * |  |  |  |
|  |  |  | **Benzenes** |  |  |  |  |  |  |  |  |  |  |
| 297.152 | 11.73 | C16H26O3S | Decylbenzenesulfonic acid | 183.05, 119.06 |  |  |  | * |  | * | * |  | * |
| 325.184 | 13.20 | C20H26N2O2 | Hydroquinidine | 309.12, 184.03, 160.04 |  |  |  |  | * | * |  | * | * |
| 325.184 | 13.10 | C22H46O | Docosanol | 279.45, 183.05, 119.06 |  |  |  | * | * | * | * | * | * |
| 339.202 | 13.60 | C21H30O2 | [5-acetyloxy-3-(hydroxymethyl)-2-oxo-6-propan-2-ylcyclohex-3-en-1-yl] 3-methylpentanoate | 123.03 |  |  |  | * | * | * | * | * | * |
| 198.13 | 6.45 | C9H10O5 | Syringic acid | 155.07, 140.06, 123.03 |  |  |  | * | * | * |  |  |  |
| 294.283 | 13.56 | C12H10N2O5S | 4-[(2,4-Dihydroxyphenyl)azo]benzenesulfonic acid | 277.67, 213.85, 155,02, 123.03 |  |  |  |  | * | * |  | * | * |
|  |  |  | **Fatty acyls** |  |  |  |  |  |  |  |  |  |  |
| 327.217 | 9.48 | C18H32O5 | Aspicilin | 211.33, 171.02, 137.05 | * | * | * | * | * | * | * | * | * |
| 328.221 | 9.34 | C18H32O5 | Malyngic acid | 183.03 |  |  |  |  | * | * | * | * | * |
| 329.233 | 9.64 | C18H34O5 | Pinellic acid | 212.64, 172.03, 127.08 | * | * | * | * | * | * | * | * | * |
| 293.175 | 9.98 | C18H30O3 | 13-Hydroxy-6,9,11-octadecatrienoic acid | 221.35, 205.06, 148.56 |  |  | * | * | * | * | * | * | * |
| 549.258 | 7.63 | C24H40O11 | 2-Cyclohexen-1-one, 3,5,5-trimethyl-4-[3-[(6-O-beta-D-xylopyranosyl-beta-D-glucopyranosyl)oxy]butyl]- | 503.08, 371.34, 161.04, 149.03 |  |  | * |  |  | * | * | * | * |
| 328.486 | 9.46 | C19H36O4 | 2,4-dihydroxyheptadec-16-enyl acetate | 283.23, 211.14, 167.04, 125.05 |  |  |  |  |  |  | * | * | * |
|  |  |  | **Flavonoids** |  |  |  |  |  |  |  |  |  |  |
| 901.238 | 7.45 | C42H46O22 | Isovitexin 2''-O-[4-hydroxy-(E)-cinnamoyl-(->6)-beta-D-glucopyranosyl] 4'-O-beta-D-glucopyranoside | 739.14, 595.02, 471.04. 429.13, 163.06, 145.05 |  |  |  |  | * |  |  | * |  |
| 905.262 | 6.41 | C56H42O12 | Vitisin C | 801.43, 679.10, 433.34, 245.08 |  |  |  |  | * |  |  | * |  |
| 961.262 | 8.17 | C44H50O24 | Isovitexin 7-(6'''-sinapoylglucoside) 4'-glucoside | 931.32, 739.03, 595.06, 429.12, 169.05, 163.06 |  |  |  | * |  |  | * |  |  |
| 769.143 | 7.01 | C34H42O20 | Typhaneoside | 315.03 |  |  |  |  |  | * |  |  |  |
| 269.045 | 8.11 | C15H10O5 | 7,3',4'-Trihydroxyflavone | 213.06, 161.02, 133.03 |  |  |  | * | * | * |  | * | * |
| 283.027 | 8.60 | C16H12O5 | Acacetin | 268.04, 239.03, 107.05 |  |  |  | * |  | * | * |  | * |
| 637.382 | 8.45 | C28H32O14 | Acacetin-7-O-rutinoside | 609.08, 283.03, 268.04 |  |  |  |  | * |  |  | * |  |
| 299.056 | 9.13 | C16H12O6 | Chrysoeriol - 40eV | 253.03, 169.05 |  |  |  |  |  |  |  |  | * |
| 595.301 | 7.48 | C27H30O15 | Datiscetin-3-O-rutinoside | 285.04, 119.06, 125.08 |  |  |  | * | * | * |  | * |  |
| 463.087 | 7.16 | C21H20O12 | Isoquercitrin | 301.05, 271.15, 243.09 |  |  |  |  | * |  | * | * |  |
| 314.466 | 7.91 | C16H12O7 | Isorhamnetin | 300.07, 285.04, 125.08 |  |  |  | * | * | * | * | * | * |
| 477.104 | 7.62 | C22H22O12 | Isorhamnetin-3-O-glucoside | 315.03 |  |  |  |  | * |  |  | * |  |
| 285.041 | 9.14 | C15H10O6 | Kaempferol | 164.04, 119.06 |  |  |  | * | * | * | * | * | * |
| 593.150 | 6.911 | C27H30O15 | Kaempferol 3-O-[2''-O-(glucopyranoside)]-rhamnopyranoside | 433.08, 287.05, 119.06 |  |  |  |  | * |  |  | * |  |
| 447.084 | 7.63 | C21H19O11 | Kaempferol-3-O-glucoside | 284.04, 285.04 |  |  |  |  | * |  |  | * |  |
| 739.209 | 7.01 | C33H40O19 | Kaempferol 3-O-(2'',6''-di-O-glucopyranoside)-glucopyranoside | 447.08, 285.04, 164.03, 119.06 |  |  |  |  | * |  |  | * |  |
| 739.225 | 8.94 | C33H40O19 | Kaempferol-3-O-robinoside-7-O-glucoside | 594.06, 449.06, 432.45, 285.04, 119.06 |  |  |  |  | * |  |  | * |  |
| 593.147 | 7.47 | C27H30O15 | Kaempferol-3-O-rutinoside | 285.04 |  |  |  | * | * | * | * | * | * |
| 594.015 | 7.41 | C27H31O15 | Keracyanine | 417.15, 205.05, 189.14, 103.03 |  |  |  | * | * | * | * | * | * |
| 447.085 | 7.87 | C21H20O11 | Luteolin-7-O-glucoside | 245.09, 287.02, 153.07 |  |  |  | * | * |  |  | * | * |
| 301.051 | 8.29 | C15H10O7 | Quercetin | 151.06, 179.04 |  |  |  |  |  | * |  |  | * |
| 771.199 | 6.07 | 33H40O21 | Quercetin glucosyl rutinoside | 609.08, 462.03, 301.05 |  |  |  |  |  |  |  |  | * |
| 463.343 | 7.08 | C12H20O12 | Quercetin hexose | 300.07 |  |  |  | * | * | * | * | * | * |
| 433.102 | 7.299 | C20H18O10 | Quercetin-O-xylofuranoside | 301.05, 229.13, 149.06 |  |  |  | * | * | * | * | * | * |
| 417.158 | 7.65 | C20H18O10 | Kaempferol-3-O-arabinoside | 284.04, 255.06 |  |  |  | * |  |  | * | * |  |
| 623.162 | 7.13 | C28H32O16 | isorhamnetin-3-O-galactoside-6''-rhamnoside | 315.03 |  |  |  | * | * | * | * | * | * |
| 272.062 | 6.37 | C15H12O5 | Naringenin | 165.03, 107.01 |  |  |  | * | * |  |  | * | * |
| 269.037 | 13.16 | C15H10O5 | Apigenin | 182.04, 118.04, 117.03 |  |  |  |  | * |  |  | * |  |
| 270.064 | 12.38 | C15H10O5 | Baicalein | 251.13, 241.14, 223.09 |  |  |  | * | * |  |  |  |  |
| 433.083 | 7.34 | C20H18O11 | Quercetin-3-O-Arabinoside | 301.05, 208.02, 225.06, 283.02 |  |  |  |  | * |  | * | * | * |
| 609.081 | 7.09 | C27H30O16 | Rutin | 301.05 |  |  |  | * | * | * | * | * | * |
| 737.826 | 8.73 | C33H40O19 | Robinin | 595.20, 433.23, 285.04 |  |  |  | * | * | * | * | * | * |
| 593.154 | 6.34 | C27H30O15 | Vicenin-2 (Apigenin 6,8-di-C- glucoside | 473.32, 383.54, 353.70 |  |  |  | * | * | * | * | * | * |
| 431.116 | 6.89 | C21H20O10 | Vitexin (Apigenin-8-C-glucoside) | 284.04, 255.06, 158.13 |  |  |  | * | * | * | * | * | * |
| 769.223 | 7.18 | C34H42O20 | Xanthorhamnin | 314.06 |  |  |  | * |  | * | * |  | * |
| 801.221 | 7.87 | C38H41O19 | Malvidin 3-(6-coumaroylglucoside) 5-glucoside | 737.40, 577. 36, 189.23, 161.05 |  |  |  |  | * |  |  | * |  |
|  |  |  | **Furanoid lignans** |  |  |  |  |  |  |  |  |  |  |
| 913.444 | 10.76 | C45H70O19 | 3b-Pregnadienolone 3-[rhamnosyl-(1->4)-rhamnosyl-(1->4)-rhamnosyl-(1->4)-glucoside] | 825.39, 649.37, 475.27, 285,295.21, 163.06 |  |  |  | * |  | * |  |  | * |
|  |  |  | **Glycerophospholipids** |  |  |  |  |  |  |  |  |  |  |
| 722.164 | 12.22 | C33H56O14 | Glc-Glc-octadecatrienoyl-sn-glycerol (isomer 2) (PUT) - 45.8016eV | 676.43, 397.27, 278.34, 179.06 |  |  |  | * | * | * | * | * | * |
| 560.025 | 12.32 | C33H56O14 | Glc-octadecatrienoyl-sn-glycerol (isomer 1) (PUT) - 39.7246eV | 278.25, 253,33, 179.06, 163,06 |  |  |  | * |  |  |  |  |  |
| 676.003 | 11.98 | C33H56O14 | Gingerglycolipid A | 397.14, 383.12,179.06, 161.05, 133.04., 103.04 |  | * |  |  |  |  |  |  |  |
| 743.506 | 14.48 | C40H75O10P | Phosphatidyl glycerol | 507.36, 279.14, 253.33, 152.10 |  |  |  | * | * | * | * | * | * |
| 571.289 | 13.68 | C25H49O12P | 1-Hexadecanoyl-sn-glycero-3-phospho-(1'-myo-inositol) | 391.35, 315.04, 255.09, 152.10 |  |  |  | * | * | * | * | * | * |
| 483.272 | 13.63 | C22H45O9P | 1-Hexadecanoyl-sn-glycero-3-phospho-(1'-sn-glycerol) | 255.09, 152.10 |  | * |  | * | * | * | * | * | * |
| 433.261 | 13.45 | C21H41O7P | lysophosphatidic acid | 433.25, 255.09, 152.10 |  |  |  | * | * | * | * | * |  |
| 375.333 | 11.81 | C33H56O14 | Linoleoyldigalactopyranosyl glycerol | 277.23, 255.10 |  |  |  | * | * | * | * | * |  |
| 859.415 | 14.38 | C55H102O6 | [3-(hexadecanoyloxy)-2-[octadec-9-enoyloxy]propoxy]phosphonic acid | 689.78, 152.09 |  | * |  | * | * | * | * | * |  |
| 961.610 | 15.83 | C49H88O15 | Glycerol 2-(9Z,12Z-octadecadienoate) 1-hexadecanoate 3-O-[alpha-D-galactopyranosyl-(1->6)-beta-D-galactopyranoside] | 917.61, 383.15, 281.25, 163.06, 103.04 |  |  |  | * | * | * | * | * | * |
| 981.579 | 14.59 | C45H74O10 | 1,2-Di-(9Z,12Z,15Z-octadecatrienoyl)-3-(Galactosyl-alpha-1-6-Galactosyl-beta-1)-glycerol | 937.59, 813.46, 383.15, 163.06, 103.04 |  |  |  | * | * | * | * | * |  |
| 915.604 | 15.83 | C49H88O15 | Glycerol 2-(9Z,12Z-octadecadienoate) 1-hexadecanoate 3-O-[alpha-D-galactopyranosyl-(1->6)-beta-D-galactopyranoside] | 897.59, 515.32, 383.28, 265.09, 179.06, 161.05, 133.05, 103.04 |  |  |  | * | * | * | * | * |  |
| 254.732 | 4.12 | C9H18O8 | 3-beta-D-Galactosyl-sn-glycerol | 235.08, 179.06,161.05, 149.05 |  |  |  | * | * | * |  |  |  |
|  |  |  | **Organooxygen compounds** |  |  |  |  |  |  |  |  |  |  |
| 461.165 | 6.50 | C19H28O10 | Phenylethyl primeveroside | 415.16, 265.09, 163.07, 147.03 |  |  |  | * | * | * | * | * |  |
| 503.138 | 3.77 | C18H32O16 | Raffinose | 485.15, 323.09, 179.06, 163.06, 147.03 |  |  |  | * | * | * | * | * |  |
|  |  |  | **Saponins** |  | |  |  |  |  |  |  |  |  |
| 955.472 | 12.33 | C51H72O17 | Tragopogonsaponin C | 937.46, 779.42, 629.37, 453.34, 147.03, 117.02 |  |  |  | * | * | * | * | * |  |
| 971.484 | 10.69 | C48H76O20 | 28-Glucosylarjunolate 3-[rhamnosyl-(1->3)-glucuronide] | 953.47, 807.41, 649. 40, 163.06, 161.05, 133.05 |  |  |  | * | * | * | * | * |  |
| 985.461 | 8.83 | C48H74O21 | 28-Glucosyl-30-methyl-3b,23-dihydroxy-12-oleanene-28,30-dioate 3-[arabinosyl-(1->3)-glucuronide] | 955.45, 853.42, 805.40, 659.38, 161.05, 133.05 |  |  |  |  |  |  | * |  |  |
| 987.479 | 9.43 | C48H76O21 | Phytolaccasaponin B | 957.47, 645.36, 179.06, 161.05, 133.05 |  |  |  |  | * |  |  |  | * |
| 883.469 | 10.63 | C45H72O17 | Deltonin | 867.16, 609.31, 383.12, 163.06, 147.07 |  |  |  | * | * |  |  |  |  |
| 897.572 | 12.25 | C45H73NO14 | alpha-Chaconine | 850.49, 704.46, 413.55, 179.06, 163.06, 119.04 |  |  |  |  | * |  | * | * |  |
| 339.199 | 13.82 | C22H28O3 | Canrenone | 311.16, 285.19, 179.10, 133.05 |  |  |  | * |  | * | * | * |  |
| 765.406 | 12.41 | C38H58O13 | Arvenin II | 719.19, 573.16, 179.06, 161.05, 119.04 |  |  |  |  | * |  |  | * | * |
| 899.478 | 9.33 | C45H72O18 | 26-Desglucoavenacoside A | 811.41, 753.41, 429. 30, 179.06, 163.06, 161. 05, 133,05 | * |  |  |  |  | * |  |  | * |
| 593.442 | 16.32 | C33H54O8 | Asparagoside A | 575.43, 465.40, 179.06, 133.05 |  |  |  | * | * | * | * | * | * |
| 595.251 | 9.05 | C33H56O9 | Asparagoside B | 565.37, 415.32, 299.24, 179.06, 161.05, 133.05 |  |  |  |  |  |  | * |  |  |
| 740.567 | 23.62 | C39H64O13 | Asparagoside C | 612.36, 415.32, 295.10, 179.06,161.05, 133.05 |  |  |  | * | * | * | * | * | * |
| 901.480 | 10.13 | C45H74O18 | Asparagoside D | 883.47, 415.32,397.31, 179.06, 161.05, 133.05 |  |  |  |  |  | * |  | * | * |
| 919.235 | 18.90 | C45H76O19 | Asparagoside E | 901.48, 625.36, 577.38, 179.06, 161.05, 133.05 |  |  |  | * | * | * | * | * | * |
| 740.567 | 23.61 | C39H64O13 | Asparanin A | 577.38, 399.13, 161.05, 133.05 |  |  |  | * | * | * | * | * | * |
| 887.419 | 20.83 | C45H74O17 | Asparanin B | 781.23, 649.14, 415.32. 273.46. 133.05 |  |  |  |  |  |  |  |  | * |
| 857.265 | 21.13 | C44H72O16 | Asparanin C | 723.33, 649.14, 415.32, 161.05, 133.05 |  |  |  | * |  | * |  |  |  |
| 911.478 | 8.34 | C50H72O15 | Tragopogonsaponin K | 803.44, 585.38, 453.34, 179.06, 163.04, 161.05, 133.05 |  |  |  | * | * | * | * | * |  |
| 913.502 | 9.67 | C50H74O15 | Tragopogonsaponin L | 895.48, 585.38, 453.34, 179.06, 161.05, 147.04,133.05 | * |  |  |  |  |  | * |  | * |
| 925.459 | 9.55 | C50H70O16 | Tragopogonsaponin B | 863.46, 629.37, 453.34, 163.04. 147.03, 117.02 |  |  |  | * | * |  | * |  |  |
| 647.465 | 25.93 | C36H56O10 | Tragopogonsaponin A | 585.38, 425.34, 163.02, 147.03,117.02 |  |  |  | * | * | * |  | * |  |
| 927. 474 | 10.73 | C47H74O18 | Chikusetsusaponin IV | 925.34, 899.54, 767,50, 443.39, 163.06, 133.05 |  |  |  | * | * |  | * |  |  |
| 943.509 | 9.33 | C51H76O16 | Tragopogonsaponin M | 913.49, 453.34, 179.03, 161.05, 133.05 |  |  |  | * | * |  | * | * | * |
| 913.053 | 11.23 | C46H72O18 | Medicagenic acid 28-O-[b-D-xylosyl-(1->4)-a-L-rhamnosyl-(1->2)-a-L-arabinosyl] ester | 877.46, 503.34, 163.06, 147.07, 133.05, 117.05 |  |  |  | * | * |  | * |  |  |
| 809.394 | 9.09 | C41H62O16 | Betavulgaroside VII | 747.40, 629.37, 427.36, 179.02, 161.01 |  |  |  | * | * |  | * |  |  |
| 837.383 | 9.38 | C42H62O17 | Licoricesaponin G2 | 352.06, 193.03, 175.02 |  |  |  | * | * |  | * |  |  |
| 865.389 | 8.64 | C43H62O18 | 25-Acetyl-6,7-didehydrofevicordin F 3-[glucosyl-(1->6)-glucoside] | 823.37, 523.27, 179.06, 161.04, 133.05, 127.08 |  |  |  | * |  |  | * |  |  |
| 933.470 | 8.31 | C45H74O20 | Ampeloside Bs1 | 915.46, 609.36, 429.30, 161.05, 133.05 |  |  |  |  | * |  |  | * | * |
| 800.485 | 14.93 | C42H72O14 | Majoroside F2 | 781.47, 637.43 ,545.38, 499.39, 161.05, 133.05 |  |  |  | * | * |  | * |  | * |
| 737.318 | 15.59 | C39H62O13 | Melongoside F | 723.43, 163.06, 161.05, 133.05 |  |  |  | * |  | * |  |  | * |
| 887.244 | 24.34 | C45H74O17 | Melongoside G | 811.45, 723.43, 163.06, 161.05, 133,05 |  |  |  |  |  |  |  |  | * |
| 885.411 | 14.24 | C45H72O17 | Melongoside H | 809.43, 629,37, 163.06, 161.05, 133.05 |  |  |  |  | * |  |  | * |  |
| 722.647 | 11.22 | C39H62O12 | Progenin III | 575.49, 395.23, 163.06, 161.04, 147.07, 133.04 |  |  |  | * | * | * | * | * | * |
| 645.182 | 19.87 | C32H38O14 | 3'-Prenylapigenin 7-[rhamnosyl-(1->6)-glucoside] | 629.22, 585.20, 205.07, 147.07, 117.05 |  |  |  |  |  |  | * | * |  |
| 956.493 | 12.31 | C48H76O19 | Elatoside I | 911.50, 749.45, 685.39, 179.06, 161.04, 133.05 |  |  |  | * | * | * | * | * |  |
| 803.446 | 10.50 | C39H66O14 | (25S)-26-O-β-D-glucopyranosyl-5β-furstan-3β, 22α, 26-triol-3-O-β-D-glucopyranoside | 757.44, 473.73 |  |  |  | * | * | * | * | * | * |
| 977.492 | 7.84 | C46H76O19 | 3-O-β-D- glucopyranosyl (1 → 2)-β-D-glucopyranosyl -22α-methoxy- (25S)—5-ene-furostane-3β,26-d | 931.49, 757.44, 595.39, 301.23 |  |  |  | * | * | * | * | * | * |

^1^ *A. falcatus* ethyl acetate

^2^ *A. plamosus* ethyl acetate

^3^ *A. densiflorus* ethyl acetate

^4^ *A. falcatus* methanol

^5^ *A. plamosus* methanol

^6^ *A. densifloru* methanol

^7^ *A. falcatus* chloroform

^8^ *A. plamosus* chloroform

^9^ *A. densiflorus* chloroform
